# A CRISPRi Gene Regulation System for Bifidobacteria

**DOI:** 10.1111/1751-7915.70260

**Published:** 2025-11-06

**Authors:** Lisa Friess, Douwe van Sinderen, Ciaran Lee

**Affiliations:** ^1^ APC Microbiome Ireland, University College Cork Cork Ireland; ^2^ School of Microbiology, University College Cork Cork Ireland; ^3^ School of Biochemistry and Cell Biology, University College Cork Cork Ireland

**Keywords:** bifidobacteria, CRISPR interference, gut microbiota

## Abstract

This work describes the development of a CRISPR interference (CRISPRi) system for targeted gene repression in bifidobacteria. We first validated the CRISPRi‐based approach using *Bifidobacterium breve* strains engineered to express nuclease‐dead orthologs of Cas9 and demonstrated that the CRISPR‐Cas system from *Streptococcus thermophilus* is efficient at targeting both reporter and endogenous genes through the use of single guide RNAs corresponding to the gene of interest. We also developed a one‐plasmid system for targeted gene repression in bifidobacteria and demonstrated its utility by targeting genes involved in nucleotide metabolism and carbohydrate metabolism in several species of bifidobacteria. Efficient gene repression was achieved across all tested bifidobacterial species without the requirement for extensive optimization of transformation parameters or sequence optimization to avoid restriction modification systems thus removing the key barriers to genetic manipulation in this genus. This CRISPRi system provides a novel approach to functional genomics in bifidobacteria which facilitates future mechanistic studies in these commercially important microbes.

## Graphical abstract

We report the development of a single plasmid‐based CRISPR interference system based on a catalytically dead Cas9 from *Streptococcus thermophilus* for targeted gene repression in bifidobacteria. This system is capable of repressing genes in a diverse range of bifidobacteria enabling functional genomics studies in non‐model strains.

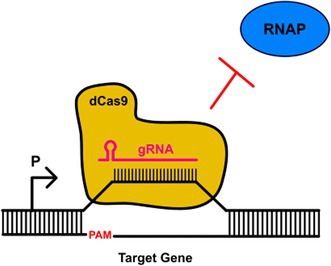

## 1 Introduction

Our bodies are home to a staggeringly large collection of microbes, in particular the human gastrointestinal tract which contains up to 0.2 kg of microbial mass (Sender et al. 2016). In healthy individuals the interplay between the immune system and the microbiota prevents tissue‐damaging inflammatory responses towards commensal bacterial species, while permitting appropriately measured immune responses against infectious microbes (Belkaid and Hand 2014). Particular species of the genus *Bifidobacterium* represent core members of commensal gut bacteria that are identified soon after birth as early colonisers of the human gastrointestinal tract (Yatsunenko et al. 2012), and remain present through all stages of life although their relative abundance declines with age (Voreades et al. 2014; Arboleya et al. 2016). These Gram‐positive, obligate anaerobes have been reported to elicit a range of beneficial effects on their host (Tojo et al. 2014; O'Callaghan and van Sinderen 2016) including immune system development and modulation (O'Neill et al. 2017), protection against pathogens (Fukuda et al. 2011), breakdown of indigestible dietary carbohydrates (Pokusaeva et al. 2011; Friess et al. 2024), and reduced symptoms of inflammatory bowel disease (Venturi et al. 1999).

Despite the purported positive impact of bifidobacteria on our health and their consequent commercial exploitation as probiotics, our current knowledge on the impact of these microbes on human health is based almost exclusively on descriptive and correlative studies with a pressing need for mechanistic studies (Fischbach 2018). In many instances, the evidence for the efficacy of probiotics in treating disease is often conflicting and confusing (Suez et al. 2019). This disconnect demonstrates that a thorough investigation of the underlying mechanisms beneficial to the host is needed to better translate research findings to inform the incorporation of bifidobacteria in commercially available functional foods and for their use as clinical interventions with a known mode‐of‐action.

While the increasing availability of bifidobacterial genome sequences allows for comparative genome analyses, a functional genomics approach is more suitable to determine gene functions and molecular mechanisms underlying phenotypic traits of interest. However, bifidobacteria are notoriously recalcitrant to genetic manipulation which has hindered the development of molecular tools for functional genomics in this genus (Brancaccio et al. 2013). This is largely due to the presence of extensive and diverse restriction/modification systems (Bottacini et al. 2018), a thick cell wall and exopolysaccharides, and sensitivity to oxygen. Targeted mutants are usually generated via site‐directed insertional mutagenesis (Hirayama et al. 2012; Sakaguchi et al. 2012; Wei et al. 2012; Hidalgo‐Cantabrana et al. 2015; O'Callaghan et al. 2015; Rizzo et al. 2024) which involves the interruption of a targeted gene with a sequence of exogenous DNA, using homologous recombination as the underlying mechanism using a Campbell‐like recombination or double crossover approach (de Vos and Simons 1994; Mercenier et al. 1994; Chopin et al. 1989; Campbell 1962). Generally, this is achieved using a conditionally replicating plasmid containing a DNA fragment corresponding to an internal part of the gene of interest and an antibiotic selectable marker gene to compensate for the low rate of homologous recombination. To overcome the low rate of homologous recombination a high transformation rate is also required; however, the composition and thickness of the bifidobacterial cell wall hinders the delivery of plasmid DNA to cells, while the presence of diverse restriction‐modification systems (Sakaguchi et al. 2012; O'Connell Motherway et al. 2009) leads to the removal of this incoming foreign DNA. This results in sub‐optimal transformation rates even with optimised protocols (Serafini et al. 2012; Park, Park, and Ji 2019). Despite these obstacles, targeted mutants have been successfully made in a limited number of strains (Hirayama et al. 2012; Sakaguchi et al. 2012; Wei et al. 2012; Hidalgo‐Cantabrana et al. 2015; O'Callaghan et al. 2015). One prominent example is *Bifidobacterium breve* UCC2003 where multiple genes have been knocked out using an insertion vector pORI19‐tet which must be propagated in a methylase‐positive *E. coli* strain expressing a cloned *B. breve* UCC2003 methylase (O'Connell Motherway et al. 2009), or through the use of a novel plasmid pFREM28 engineered to be devoid of any *B. breve* R‐M recognition sequences (Hoedt et al. 2021). Both approaches are laborious and time‐consuming and require prior knowledge of active R‐M systems within the to‐be‐targeted bifidobacterial strain.

The genetic engineering toolkit has expanded massively over recent years with the development of CRISPR‐based tools. These tools have been adapted for use in a broad range of organisms from *E. coli* to humans (Qi et al. 2013; Butt et al. 2017; Jarrett et al. 2018; Park, Lee, et al. 2019). The CRISPR‐Cas (clustered regularly interspaced short palindromic repeats‐CRISPR‐associated proteins) systems function as adaptive immune systems in a wide range of prokaryotes providing protection against foreign nucleic acids (Mojica et al. 2005; Barrangou et al. 2007). Despite the widespread use of CRISPR‐Cas in diverse species its adoption in *Bifidobacterium* species has been rather limited. The endogenous type I‐G CRISPR‐Cas system in *B. animalis* subsp. *lactis* ( *B. lactis* ) (Pan et al. 2022) and the endogenous type I‐C CRISPR‐Cas system in *B. breve* (Han et al. 2024) have been repurposed for mutagenesis. Editing with an exogenous CRISPR‐Cas system has been achieved in *B. animalis* (Li et al. 2023). These studies relied upon homologous recombination with CRISPR‐Cas, providing counter‐selection, limiting its application to strains that can easily be transformed and in the case of endogenous systems to strains containing the respective type I CRISPR‐Cas system. Cytosine base editors have been used in *B. lactis* allowing for targeted modification of cytosine to thymidine although variation in efficiency and intended base editing was observed across different strains of *B. lactis* (Pan et al. 2022).

CRISPR interference (CRISPRi) uses a catalytically inactive (referred to as dead) Cas9 (dCas9) to repress the expression of a target gene of interest (Qi et al. 2013; Bikard et al. 2013). CRISPRi can target virtually any genomic sequence through designer single guide RNAs (sgRNAs) which recruit dCas9 to bind to the non‐template strand of a target gene where it acts as a roadblock for RNA polymerase resulting in targeted transcriptional repression of the target gene (Qi et al. 2013; Bikard et al. 2013). Here we demonstrate that CRISPRi is functional in bifidobacteria and develop a one‐plasmid CRISPRi system for use in various *Bifidobacterium* species. Using this system, we demonstrate targeted repression of nucleotide metabolism, exopolysaccharide production, and carbohydrate metabolism. CRISPRi‐induced phenotypes were generated without the requirement for high transformation efficiencies. This study adds CRISPRi to the genetic engineering toolkit for bifidobacteria and provides a basis for further development of genome engineering tools based on CRISPRi for a deeper understanding of the molecular mechanisms underlying their beneficial properties.

## 2 Materials and Methods

### 2.1 Bacterial Strains and Culture Conditions

*Escherichia coli* strains were routinely cultured in Luria‐Bertani (LB) broth at 37°C with agitation at 200 rpm. Bifidobacterial strains were routinely cultured under anaerobic conditions in a Modular Atmosphere Controlled System (Davidson and Hardy Ltd., Dublin, Ireland) at 37°C in modified de Man Rogosa Sharpe (mMRS) medium (de Man et al. 1960; Watson et al. 2013) prepared from first principles and supplemented with 1% glucose and 0.05% cysteine‐HCl with the exception of *B. longum* subsp. *longum* strains which were cultured in mMRS with 1% lactose and 0.05% cysteine‐HCl. Where appropriate, alternative carbon sources were used at a final concentration of 1% (w/v) and growth medium was supplemented with ampicillin (Amp; 100 μg/mL for *E. coli* ), erythromycin (Em; 250 μg/mL for *E. coli* , 150 μg/mL for *B. breve* ), chloramphenicol (Ch; 25 μg/mL for *E. coli* , 10 μg/mL for *B. breve* ) or 5‐fluorouracil (0.1–5 μM) (Sigma‐Aldrich, Steinheim, Germany). All bacterial strains used in this study are listed in Table S1. For CRISPRi growth assays in *B. breve* and *B. animalis* subsp. *animalis*, strains were cultured in 200 μL of media in a 96‐well plate reader with OD_620nm_ readings taken every 30 min. For CRISPRi growth assays in *B. longum* subsp. *infantis* and *B. longum* subsp. *longum*, strains were cultured in 5 mL static cultures under anaerobic conditions with periodic measurement at OD_600nm_.

### 2.2 Generation of *B. breve* UCC2003 Strains Containing dCas9

A 500 bp fragment of the native CRISPR locus from *B. breve* UCC2003 (position 1,747,801–1,748,301 of the deposited genome sequence (accession number: PRJNA13487)) was amplified by PCR using *B. breve* UCC2003 genomic DNA as a template. All primer sequences used in this study are listed in Table S2. The generated PCR products were ligated into pFREM28 (Hoedt et al. 2021) using AatII and NotI (New England Biolabs, Ipswich, MA, US). The coding sequences of catalytically dead Cas9 (dCas9) from *Streptococcus pyogenes* and *Streptococcus thermophilus* were codon optimized for expression in *B. breve* and designed to exclude restriction modification motifs present in *B. breve* (Bottacini et al. 2018) prior to being chemically synthesized (Twist Biosciences, San Francisco, CA, US) and cloned into pFREM28 using AatII and XhoI (New England Biolabs). A codon optimized BetI transcriptional repressor‐encoding gene was also cloned into pFREM28 using EcoRI and XhoI (New England Biolabs) resulting in dCas9 expression being under the control of a pBet promoter. The resulting pFREM28‐betI‐dCas9 plasmids were cloned and propagated in *E. coli* EC101 which contains the required replicase‐encoding gene for plasmid replication (Law et al. 1995). All plasmids used in this study are listed in Table S3. *B. breve* UCC2003 was transformed as previously described with minor adaptations (Fanning et al. 2012). Briefly, a single colony was grown in mMRS supplemented with 1% glucose and 0.05% cysteine under anaerobic conditions at 37°C for 16 h, subcultured and grown until an OD_600nm_ of 0.4–0.6 was reached. Cells were harvested by centrifugation (10 min at 4500 × *g*) and washed twice with 0.5 M sucrose in 1 mM citrate buffer before a final resuspension in 200 μL of 0.5 M sucrose in 1 mM citrate buffer. Competent cells were electroporated (2.5 V, 20 kW, 300 Ω) and recovered in Reinforced Clostridium Medium (RCM) (Oxoid, Hampshire, UK) for 2.5 h at 37°C, plated on RCA with 150 μg/mL of erythromycin for 48 h. PCR was used to validate the insertion of dCas9 constructs into the native CRISPR locus and subsequently corroborated by DNA sequencing of the PCR products to confirm the genome‐insertion boundaries (Genewiz, Leipzig, Germany).

### 2.3 Generation of *B. breve* UCC2003 dCas9 Strains Containing Nanoluciferase

An alternative integrating vector for *B. breve* pFREM29 was created by replacing the origin of replication in pFREM28 with a modified ColEI origin. The erythromycin resistance marker was then replaced with a chloramphenicol resistance marker to generate pFREM29‐CAT. A 500 bp fragment of the integrated erythromycin resistance marker present in *B. breve* dCas9 strains was amplified by PCR and cloned into pFREM29‐CAT using NotI and XbaI (New England Biolabs) to generate pFREM29‐CAT‐E500. The coding sequence of nanoluciferase was codon optimized for expression in *B. breve* and designed to exclude restriction modification motifs present in *B. breve* UCC2003 (Bottacini et al. 2018) prior to being chemically synthesized (Twist Biosciences) and cloned into pFREM29‐CAT‐E500 with EcoRI and NotI to generate pFREM29‐CAT‐E500‐nanoluciferase. This plasmid was transformed into *B. breve* ‐dCas9 strains by electroporation and the presence of integrated nanoluciferase in resulting clones was verified by PCR and by DNA sequencing of the PCR products to confirm the genome‐insertion boundaries (Genewiz).

### 2.4 Generation of gRNA Expression Vectors

The vector pNZ000 was generated by removing the *B. breve* UCC2003 restriction modification motifs from pNZ44 (McGrath et al. 2001) using PCR mutagenesis. A gRNA expression cassette was chemically synthesised (Integrated DNA Technologies, Coralville, IA, US) and cloned into pNZ000. Plasmids were constructed containing the gRNA scaffold sequence from *S. pyogenes* and *S. thermophilus* . All gRNA expression cassettes contain two BbsI restriction sites to facilitate cloning of protospacer target sequences. Complementary gRNA oligonucleotides (Integrated DNA Technologies) were annealed, phosphorylated with T4 polynucleotide kinase (New England Biolabs), and ligated using T4 DNA ligase (New England Biolabs) into the corresponding gRNA expression vector pNZ000‐gRNA_Spy_ or pNZ000‐gRNA_Sth1_ using a BbsI‐based Golden Gate cloning reaction. gRNA targeting sequences are listed in Table S4.

### 2.5 Luciferase Assay

For each sample, overnight cultures were diluted back to OD_600nm_ of 0.1 with mMRS with or without 10 mM choline chloride to induce CRISPRi. Nanoluciferase knockdown was allowed to proceed for 4 h corresponding to the log phase of *B. breve* UCC2003. Five hundred microlitre of culture was harvested by centrifugation and processed for nanoluciferase assays as follows. Bacterial cells were pelleted by centrifugation and resuspended in 50 μL of CellLytic B lysis reagent (Sigma‐Aldrich) supplemented with 0.2 mg/mL of lysozyme (Sigma‐Aldrich) and incubated at 37°C for 15 min. 8 μL of the resulting lysate was added to 8 μL of ONE‐Glo reagent buffer in a white opaque 384‐well plate. 8 μL of Nano DLR Stop ‘N’ Glo reagent buffer (Promega, Madison, WI, US) was added to each well and incubated at room temperature with shaking for 10 min. Luciferase activity was quantified using a Synergy 2 plate reader (Biotek, Winooski, VT, US).

### 2.6 Sedimentation Assay

*B. breve* UCC2003, a Bbr_0430 mutant strain and EPS CRISPRi clones were grown in culture overnight at 37°C under anaerobic conditions. All cultures were brought into suspension via vortexing. The OD_600nm_ values were recorded, and the culture tubes were incubated vertically at room temperature without agitation or movement. At each timepoint a 200 μL sample was taken from the air‐liquid interface of the cultures and the OD_600nm_ values were measured.

### 2.7 Identification of Sth1 gRNAs in *B. breve* UCC2003

The *B. breve* UCC2003 genome was searched for all potential gRNA sequences using the open‐source tool GuideFinder (Spoto et al. 2020) with the following modifications. The permissible PAM sequence was changed to search for all functional Sth1 PAMs (Rock et al. 2017) and the RE_site filter was modified to remove gRNAs containing the *B. breve* UCC2003 sequences CTGCAG, RTCGAY, and GGCGCC (Bottacini et al. 2018). Finally, all gRNAs targeting redundant or repeat sequences were removed resulting in a list of Sth1 gRNAs with a unique target site within the *B. breve* UCC2003 genome (Table S5).

### 2.8 Generation of All‐In‐One CRISPRi Plasmids

The *S. thermophilus* dCas9 gene sequence was cloned into pBC1.2 (Álvarez‐Martín et al. 2008) under the control of the constitutive promoter J23119 (Registry of Standard Biological Parts) using NotI and BamHI (New England Biolabs) to generate the plasmid pCL007. To facilitate gRNA cloning, an intermediate *E. coli–Bifidobacterium* shuttle vector (pCL001) was created by cloning the *Bifidobacterium* origin of replication from pBC1.2 into pFREM29 containing a chloramphenicol resistance gene. A *Sth1* gRNA expression cassette containing two SapI restriction sites to facilitate cloning of gRNA spacer sequences was cloned into pCL001 to generate pCL002. For all‐in‐one gene targeting plasmids gRNA target sequences were cloned into pCL002 (Figure S1) using a SapI‐based Golden Gate cloning reaction. The resulting gRNA expression cassettes were cloned into pCL007 (Figure S2) using EcoRI and KpnI.

### 2.9 Obtaining Competent Bifidobacterial Cells for Genetic Transformation

*B. breve* , *B. longum* subsp. *infantis*, and *B. animalis* subsp. *animalis* were grown in mMRS supplemented with 1% glucose and 0.05% cysteine under anaerobic conditions at 37°C for 16 h, subcultured and grown until an OD_600nm_ of 0.4–0.6 was reached. Cells were harvested by centrifugation (10 min at 4500 × *g*) and washed twice with 0.5 M sucrose in 1 mM citrate buffer before a final resuspension in 200 μL of 0.5 M sucrose in 1 mM citrate buffer. Competent cells were electroporated (2 V, 20 kW, 200 Ω) after which *B. breve* and *B. longum* subsp. *infantis* were recovered in Reinforced Clostridium Medium (Oxoid) for 2.5 h at 37°C and plated on RCA with 10 μg/mL of chloramphenicol for 48 h. *B. animalis* subsp. *animalis* was recovered in mMRS supplemented with 1% glucose and 0.05% cysteine for 2.5 h at 37°C and plated on mMRS agar with 10 μg/mL of chloramphenicol for 48 h.

To obtain competent cells for *B. longum* subsp. *longum* NCIMB8809, this strain was grown in MRS supplemented with 7% sucrose (Fisher Scientific) and 34 μg/mL Iron (II) Sulfate Heptahydrate (Fisher Scientific) and 0.05% cysteine under anaerobic conditions at 37°C for 16 h, subcultured twice, and grown until an OD_600nm_ of 0.9–1.1 was reached, before further subculturing in MRS with the addition of 7% sucrose, 1% lactose, 0.05% cysteine and 20 mM NaCl and grown until an OD_600nm_ of 0.35–0.5 was reached. Cells were harvested by centrifugation (10 min at 4500 × *g*) and washed 3 times (1 min at 15,000 × *g*) with 0.5 M sucrose in 1 mM citrate buffer before a final resuspension in 200 μL of 0.5 M sucrose in 1 mM citrate buffer. Competent cells were electroporated (2.5 kV, 20 kW, 300 Ω) after which they were recovered in MRS supplemented with 100 ng/mL CaCl_2_ (w/v) and 50 ng/mL (w/v) 1,4‐dihydroxy‐2‐naphthoic acid (DHNA; Sigma‐Aldrich) for 3 h at 39°C, and plated on RCA with 100 ng/mL CaCl_2_ (w/v) and 50 ng/mL (w/v) DHNA and 10 μg/mL of chloramphenicol for 48 h at 39°C. Plasmids used to transform *B. longum* NCIMB8809 were propagated in an *E. coli* strain EC101‐pNZEM‐M.blmncII which contains a *B. longum* methylase (O'Callaghan et al. 2015).

### 2.10 Statistical Analysis

To assess the effects of CRISPRi on bacterial growth (OD_600nm_ measurements), a repeated measures ANOVA was used. Initially, outliers were identified, and extreme outliers were removed to prevent distortion of statistical assumptions and results. Outliers were defined as extreme if they were more than three times the inter quartile range beyond the first or third quartile. Data normality was evaluated using Shapiro–Wilk tests and if all groups did not meet the threshold for normality the data was log transformed prior to ANOVA testing. Post hoc pairwise *t*‐tests were conducted to explore growth differences between strains in each sugar with Bonferroni adjustment to reduce Type I errors. For growth curve data obtained from 96‐well plate assays (OD_620nm_ measurements) the R package Growthcurver was used (Sprouffske and Wagner 2016). Growthcurver computes the area under the logistic curve by integrating information from the following logistic parameters: *K* (carrying capacity), *r* (intrinsic growth rate), and *N*_0_ (population size at the beginning of the growth curve). The area under the curve (AUC) of growth curves in the test sugar media was normalised to the AUC of growth curves in media containing glucose. The resulting normalised AUC data was analysed by ordinary one‐way ANOVA and post hoc pairwise *t*‐tests were conducted to explore growth differences between strains in each sugar with Bonferroni adjustment to reduce Type I errors. Colony count data was analysed using *t*‐tests for independent samples of equal variance.

### 2.11 Transcriptome Analysis by RNAseq

RNAseq was performed on *B. longum* subsp. *longum* NCIMB 8809 strains expressing dCas9 or dCas9 and *axuA*‐targeting gRNAs from *n* = 2 replicates. Strains were grown overnight in mMRS with 0.06% cysteine‐HCl, 1% lactose and then subcultivated in mMRS with 0.06% cysteine‐HCl and 0.5% arabinose for 7 h. Cells were harvested by centrifugation and resuspended in 300 μL RNA shields (Zymo Research, Orange, CA, USA). The resulting cell suspensions were sent to the University of Groningen where RNA extraction, rRNA depletion, library construction and subsequent RNA sequencing were performed. To eliminate ribosomal RNA from the 250 ng total RNA, the RiboCop rRNA depletion kit (Lexogen Vienna, Austria) was employed. Subsequently, the NEBNext Ultra II Directional RNA Library Prep Kit (E7765, New England Biolabs) was utilised to prepare the library preps for Illumina sequencing. The sequencing process was performed on an Illumina NexSeq 1000 sequencing instrument, generating 100 bases single‐end reads (100SE) with an average read depth ranging between 8 and 12 million reads per sample. The quality of the resulting fastq reads was assessed using FastQC v0.11.9 (Babraham Bioinformatics, Cambridge), followed by mapping on the reference genome using Bowtie2 v2.4.2 with default settings. The resulting SAM files were converted to BAM format using SAMtools 1.11, and gene counts were obtained using featuresCounts 2.0.1 (Subread/2.0.2). Differential gene expression analysis was performed using the R statistical platform and the LIMMA package available as part of the Bioconductor release (v.3.21). As a preprocessing step lowly expressed genes were discarded prior to differential gene expression analysis. The resulting data was transformed using the voom function and genes with an adjusted *p*‐value < 0.05 and a log2‐fold change > 1.5 were considered significantly up‐ or down‐regulated.

## 3 Results and Discussion

### 3.1 dCas9 CRISPRi Systems in Bifidobacteria

**FIGURE 1:**
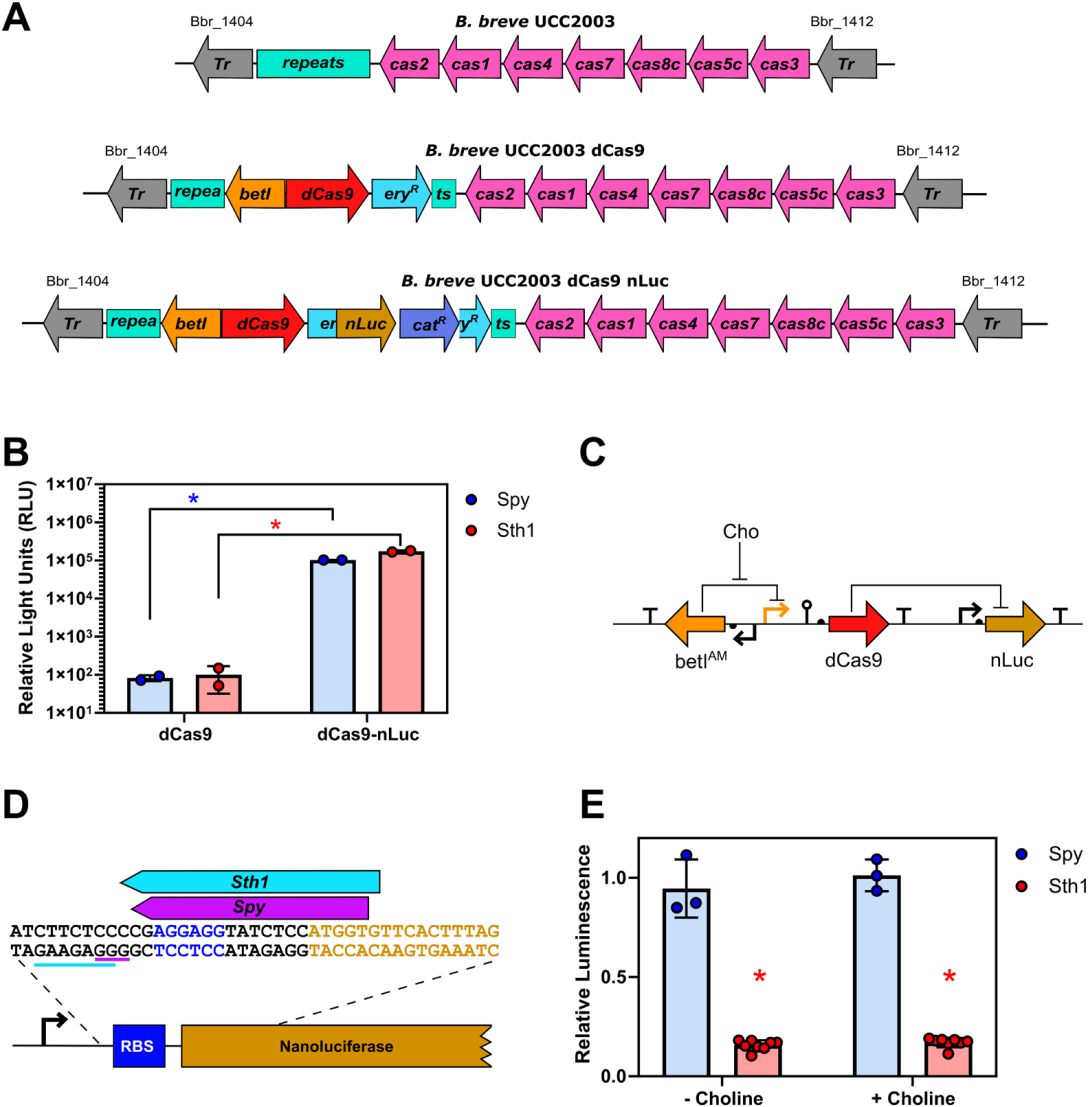
In vivo validation of functional CRISPR interference in bifidobacteria. (A) Schematic of *B. breve* UCC2003 strains with integrated CRISPRi and nanoluciferase expression systems. (B) The nanoluciferase reporter is expressed in nanoluciferase containing strains (dCas9‐nLuc) resulting in detectable luminescence as measured by luciferase assay. *N* = 2, *p < 0.001, two‐way ANOVA. (C) Genetic circuit containing a choline inducible dCas9 (P_Bet_) directed to target nanoluciferase. (D) gRNA sequences designed to target the 5′ untranslated region (UTR) of nanoluciferase. The coding sequence is shown in orange, the RBS sequence is highlighted in blue and the dCas9 PAM sequences are underlined in blue (*Sth1*) and purple (*Spy*). (E) Relative luminescence observed with gRNAs targeting the 5′ UTR of nanoluciferase compared to the non‐targeting gRNA NT1 when dCas9 is expressed at basal (− Choline) or induced (+ Choline) levels. *N* = 3 for Spy dCas9 and *N* = 8 for Sth1_dCas9, *p < 0.0001, two‐way ANOVA. Error bars represent standard deviation.

To evaluate CRISPRi systems in bifidobacteria we engineered *B. breve* UCC2003 to express a catalytically dead Cas9 from *Streptococcus pyogenes* (*Spy* dCas9) or *Streptococcus thermophilus* (*Sth1* dCas9) under the control of a choline‐inducible *betI* regulated promoter (Meyer et al. 2019; Nielsen et al. 2016; Stanton et al. 2014). The coding sequences of *Spy* dCas9, *Sth1* dCas9 and *betI* were codon optimised (Puigbò et al. 2007) and known *B. breve* restriction modification motifs (Bottacini et al. 2018) were removed from the open reading frames. The resulting sequences were synthesized and cloned into the suicide vector pFREM28 (Hoedt et al. 2021). To avoid disrupting any endogenous genes, the resulting plasmids were designed to integrate dCas9 into a non‐coding region of the native CRISPR locus of *B. breve* UCC2003 via a 500 bp region of homology generating *B. breve* UCC2003‐*Spy* dCas9 and *B. breve* UCC2003‐*Sth1* dCas9 (Figure 1A). These CRISPRi strains were further engineered using pFREM28 to integrate a codon‐optimized nanoluciferase expression cassette into the chloramphenicol resistance gene to create *B. breve* UCC2003‐*Spy* dCas9‐nLuc and *B. breve* UCC2003‐*Sth1* dCas9‐nLuc which express functional nanoluciferase (Figure 1A,B). The *betI*‐encoded regulator allows for choline‐inducible gene expression of dCas9 (Figure 1C). To test the ability of the CRISPRi systems to repress target gene expression plasmids containing *Spy* and *Sth1* gRNAs targeting the 5′ end of the non‐template strand of the nanoluciferase reporter gene were used (Rock et al. 2017) (Figure 1D), while the non‐targeting gRNA NT1 (Table S4) was used as a negative control. Repression of the nanoluciferase reporter was observed with the *Sth1* dCas9 system; however, no apparent repression of luciferase activity was observed with the *Spy* dCas9 system (Figure 1E). The superior performance of *Sth1* dCas9 is in agreement with previous studies in another GC‐rich microbe (Rock et al. 2017). Despite dCas9 being placed under the transcriptional control of a BetI‐inducible promoter repression of luciferase expression was observed in the absence of the inducer compound choline (Figure 1E), indicating that this promoter system is constitutively active in *B. breve* UCC2003. All subsequent experiments were carried out using the *Sth1* dCas9 system. The consensus Cas9_Sth1_ PAM sequence NNAGAAW presents limited targeting functionality in GC‐rich bifidobacterial genomes; however, recent studies have demonstrated that deviations within this sequence are tolerated with minimal loss of targeting efficiency (Rock et al. 2017; Esvelt et al. 2013; Kleinstiver et al. 2015; Leenay et al. 2016; Karvelis et al. 2015) resulting in 28,085 possible target sites within the *B. breve* UCC2003 genome or approximately one target site every 86 base pairs (Table S5).

### 3.2 dCas9_Sth1_ Enables Targeted Gene Knockdown of Endogenous Genes in Bifidobacteria

**FIGURE 2:**
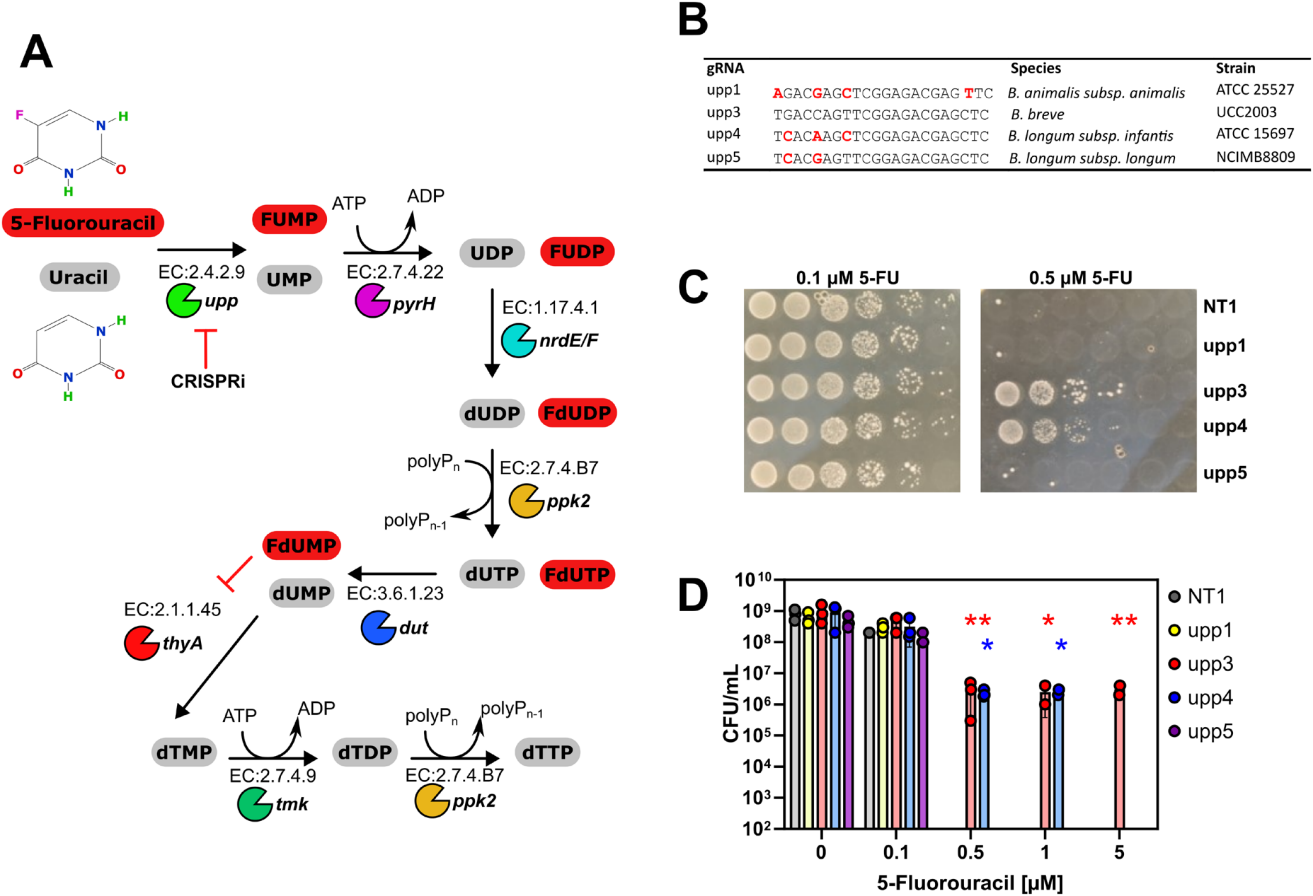
Survival of *B. breve* UCC2003 with CRISPRi repression of *upp* (A) schematic of the uracil salvage pathway in *B. breve* UCC2003 indicating metabolism of 5‐fluorouracil that leads to production of 5‐fluoro2′‐deoxyuridine‐5′‐monophosphate (FdUMP) and inhibition of thymidylate synthase (*thyA*). (B) gRNA sequences targeting orthologs of *upp* found within bifidobacteria. Mismatches with the *B. breve* UCC2003 *upp* sequence are highlighted in red. (C) The impact of *upp* repression by plating dilutions of each strain on media containing varying concentrations of 5‐fluorouracil (5‐FU). (D) Colony counts for each strain plated on 5‐FU media demonstrating increased resistance to 5‐FU toxicity with targeted gene repression. *N* = 3, *p < 0.05, **p < 0.01, two‐way ANOVA. Error bars represent standard deviation.

gRNAs targeting endogenous genes were designed to determine if native *B. breve* genes could be repressed. First, uracil phosphoribosyltransferase (*upp*), which is an essential component of the uracil salvage pathway, was targeted (Figure 2A) using gRNAs specific for *upp* in *B. breve* UCC2003, gRNAs designed to target other bifidobacterial orthologs of *upp* (Figure 2B) and the non‐targeting control gRNA NT1 5‐fluorouracil (5‐FU) is an analogue of uracil with a fluorine atom at the C‐5 position. It can enter the uracil salvage pathway after being metabolised by *upp* resulting in the production of active metabolites such as fluorodeoxyuridine monophosphate (FdUMP), which inhibits thymidine synthase (*thyA*), and fluorodeoxyuridine triphosphate (FdUTP) and fluorouridine triphosphate (FUTP), which disrupt DNA and RNA synthesis, respectively (Longley et al. 2003), resulting in inhibition of bacterial growth and reproduction. Bacterial toxicity of 5‐FU treatment is also mediated through reduced cell wall biosynthesis due to the presence of 5‐FU‐containing sugar nucleotides (Singh et al. 2015). Using growth media containing increasing concentrations of 5‐FU it was determined that the presence of 0.5 μM 5‐FU is sufficient to inhibit the growth of *B. breve* UCC2003 (Figure 2C). CRISPRi knockdown of *upp* in *B. breve* UCC2003 confers resistance to 5‐FU up to 5 μM and this high degree of resistance was dependent on a perfectly matched gRNA sequence (Figure 2C,D). Mismatch tolerance within the gRNA sequence has been observed when using CRISPR‐Cas editing tools in organisms across different kingdoms of life (Park, Lee, et al. 2019; Sturme et al. 2022; Cui et al. 2018). Similarly, we observed that a mismatched gRNA (upp4) was capable of inducing resistance to 5‐FU demonstrating that off‐target effects could result from CRISPRi targeting in *B. breve* UCC2003 (Figure 2D). However, the upp5 gRNA with fewer mismatches did not confer any resistance demonstrating that the position and nature of the mismatch also influence target specificity.

**FIGURE 3:**
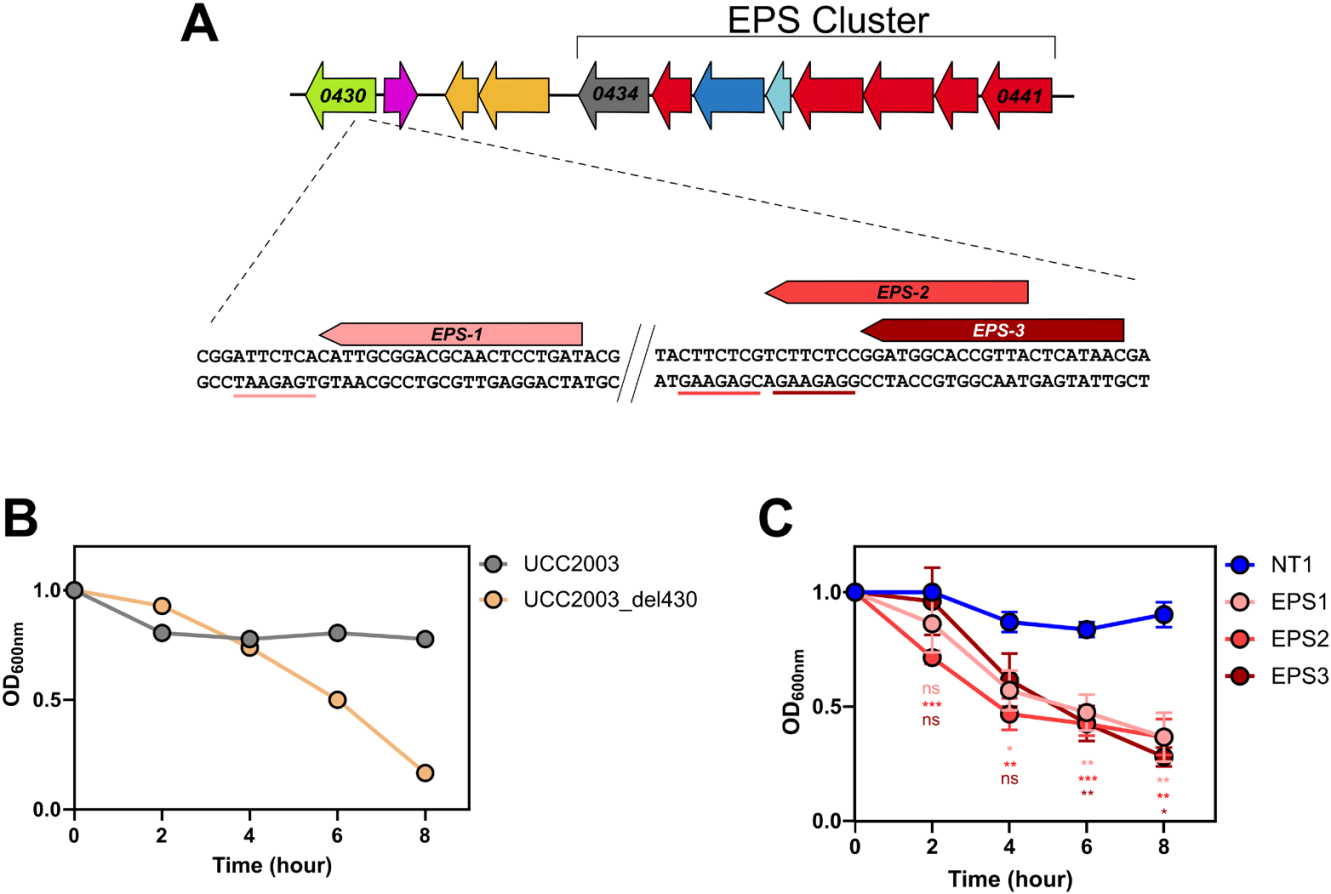
CRISPRi repression of exopolysaccharide production. (A) schematic of the major EPS locus in *B. breve* UCC2003 and the gRNA target sequences within the *Bbr_0430* open reading frame. The dCas9 PAM sequences are underlined. (B) OD measurements (OD_600nm_) of *B. breve* UCC2003 and a *Δ430* mutant strain over an 8‐h time period. The observed drop in OD values for the *Δ430* mutant strain is due to cell sedimentation. (C) OD measurements (OD_600nm_) of *B. breve* UCC2003 and strains containing one of three gRNAs targeting *Bbr_0430*. *N* = 8, *p < 0.05, **p < 0.01, ***p < 0.005, two‐way ANOVA. Error bars represent standard deviation.

Next we targeted exopolysaccharide (EPS) production in *B. breve* UCC2003 using 3 separate gRNAs targeting the priming glycosyltransferase responsible for EPS production (*Bbr_0430*) (Fanning et al. 2012) (Figure 3A). All three gRNAs target the non‐template strand of *Bbr_0430* at varying distances from the transcription start site. A previously characterised EPS‐negative mutant strain of *B. breve* UCC2003 displays a sedimentation phenotype after growth in liquid medium (Fanning et al. 2012) (Figure 3B). When *Bbr_0430* is targeted with the *Sth1* CRISPRi system the resulting loss of EPS expression results in an obvious sedimentation phenotype with all three distinct gRNAs targeting *Bbr_0430* whereas no sedimentation is observed with the non‐targeting gRNA NT1 over the time frame of the assay (Figure 3C). Successful repression of *upp* and EPS production demonstrate this CRISPRi system can be used to model both gain of function and loss of function phenotypes in *B. breve* .

### 3.3 Targeted Repression of Carbohydrate Metabolism

**FIGURE 4:**
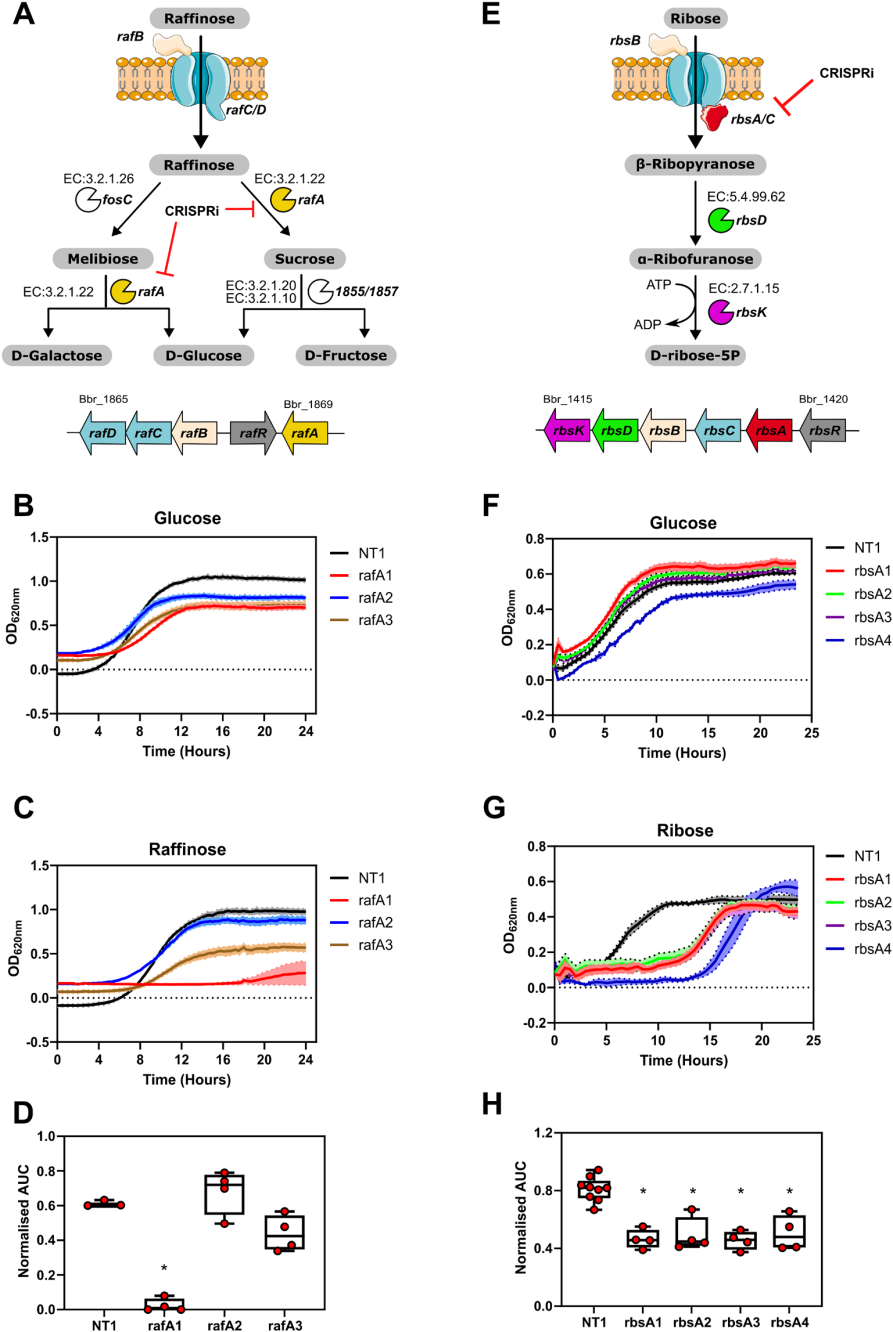
CRISPRi repression of carbohydrate metabolism in *B. breve* UCC2003. (A) Schematic of the raffinose metabolic pathway in *B. breve* UCC2003. (B) Growth of *B. breve* UCC2003 CRISPRi strains with control gRNA and gRNAs targeting *rafA* in media containing glucose. *N* = 4. Lines represent mean values, and the shaded area represents standard deviation. (C) Growth of *B. breve* UCC2003 CRISPRi strains with control gRNA and gRNAs targeting *rafA* in media containing raffinose *N* = 4. Lines represent mean values, and the shaded area represents standard deviation. (D) Normalised area under the curve (AUC) data for *B. breve* CRISPRi strains grown in the presence of glucose and raffinose. *N* = 4, *p < 0.001, whiskers represent minimum and maximum values. (E) Schematic of the ribose metabolic pathway in *B. breve* UCC2003. (F) Growth of *B. breve* UCC2003 CRISPRi strains with control gRNA and gRNAs targeting *rbsA* in media containing glucose. *N* = 4. Lines represent mean values, and the shaded area represents standard deviation. (G) Growth of *B. breve* UCC2003 CRISPRi strains with control gRNA and gRNAs targeting *rbsA* in media containing ribose. *N* = 4. Lines represent mean values, and the shaded area represents standard deviation. (H) Normalised area under the curve (AUC) data for *B. breve* CRISPRi strains grown in the presence of glucose and ribose. *N* = 4, *p < 0.001, one‐way ANOVA. Whiskers represent minimum and maximum values.

Bifidobacteria are known to metabolise a wide range of sugars and over 8% of genes identified in bifidobacteria are predicted to be involved in carbohydrate metabolism (Pokusaeva et al. 2011; Ventura et al. 2009). The availability of different carbohydrates in the human intestinal environment influences the ability of different bifidobacterial species to grow and colonise the intestinal tract which has a direct effect on bifidobacterial fermentation end product production such as acetate and lactate that are believed to impact directly and indirectly on host health (Friess et al. 2024). Given the importance of carbohydrate metabolism to Bifidobacteria we used the CRISPRi system to target genes in *B. breve* UCC2003 involved in ribose and raffinose metabolism. *Bbr_1869* (*rafA*) encodes an α‐galactosidase involved in the metabolism of raffinose, stachyose and related carbohydrates (O'Connell et al. 2014) (Figure 4A), whereas *Bbr_1419* (*rbsA*) encodes for a ribose‐specific transport system ATP‐binding protein, being involved in ribose metabolism (Pokusaeva et al. 2010) (Figure 4E). gRNAs were designed to target both genes and transformed into *B. breve* UCC2003‐dCas9_Sth1_. The resulting clones were grown in the presence of glucose, ribose, or raffinose as the sole carbohydrate source. Two of three gRNAs targeting *rafA* resulted in decreased growth (rafA3) or lack of growth (rafA1), whereas rafA2 had no impact on the growth of *B. breve* in the presence of raffinose (Figure 4C). However, the rafA CRISPRi clones also had reduced growth in the presence of glucose (Figure 4B) and only the rafA1 gRNA had a significantly reduced growth rate in the presence of raffinose when growth rates were normalised to the glucose control condition (Figure 4D). The rafA2 and rafA3 gRNAs target the template strand of the *rafA* open reading frame and are not expected to have a strong impact on gene expression (Qi et al. 2013; Rock et al. 2017). The successful repression of *rafA* with CRISPRi and the resulting phenotype, i.e., lack of bacterial growth in the presence of raffinose relative to glucose validates *rafA* as an essential component of the raffinose metabolic pathway (O'Connell et al. 2014) and demonstrates specific targeting with the *Sth1* CRISPRi system. When *rbsA* was targeted, delayed growth and an increased lag phase were observed in the presence of ribose, while a slight reduction in the growth rate was observed in the presence of glucose (Figure 4F,G). When the growth rate in the presence of ribose was normalised to the glucose control condition all four independent clones containing an *rbsA* targeting gRNA had significantly reduced growth in the presence of ribose compared to clones containing a non‐targeting gRNA sequence (Figure 4H). This carbohydrate‐specific effect demonstrates specific targeting of *rbsA* with the CRISPRi system. The reduced efficacy of targeting *rbsA* compared to *rafA* could arise due to different efficiencies of the gRNA sequences or the essentiality of each gene to its respective metabolic pathway; for example, *rbsA* is a transporter whereas *rafA* is the metabolic enzyme that acts directly on raffinose.

### 3.4 All‐In‐One CRISPRi Plasmid System for Bifidobacteria

**FIGURE 5:**
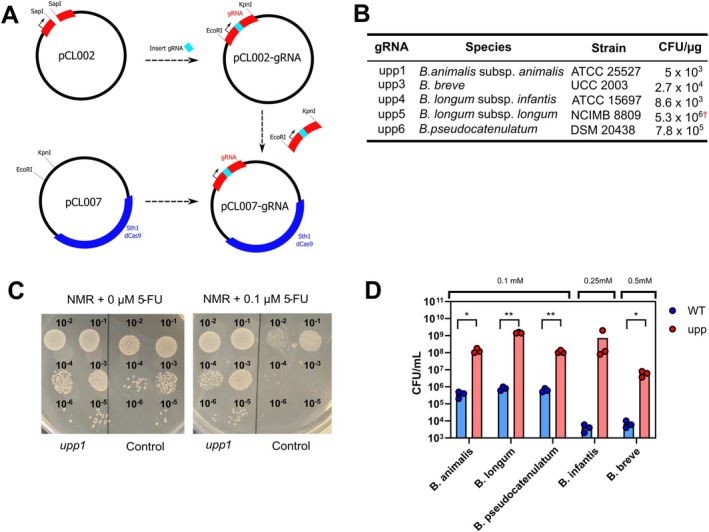
All‐in‐one plasmid system for CRISPRi in bifidobacteria. (A) Schematic of the cloning strategy to generate an all‐in‐one CRISPRi expression system in pCL007. (B) Transformation efficiency of the pCL007 plasmid in different species of bifidobacteria. † Transformation efficiency in *B. longum* NCIMB8809 is after methylation of pCL007 with B8809_607. (C) Impact of *upp* repression in *B. animalis* plated on mMRS agar containing 0 or 0.1 mM 5‐FU. (D) Colony counts for each strain plated on 5‐FU media demonstrating increased resistance to 5‐FU toxicity with targeted gene repression. The concentration of 5‐FU used for each species/strain is indicated above the data bars. *N* = 3, error bars represent standard deviation. *p < 0.01, **p < 0.001, *t*‐test.

Having demonstrated proof‐of‐concept for CRISPRi in *B. breve* UCC2003 we next focused on applying dCas9_Sth1_ for effective gene repression in other species of the genus *Bifidobacterium*. The use of nonreplicating vectors for the generation of dCas9_Sth1_ strains via homologous recombination is limited in bifidobacteria by low transformation efficiencies and the presence of diverse restriction‐modification systems. Therefore, we decided to develop an all‐in‐one CRISPRi plasmid system containing a constitutively expressed dCas9_Sth1_ and a targeting gRNA (pCL007‐gRNA) capable of achieving efficient gene repression independent of the need for high transformation efficiencies (Figure 5A). When pCL007 plasmids containing *upp* targeting gRNAs were transformed into different bifidobacterial species the transformation efficiency ranged from 5 × 10^3^ to 7.8 × 10^5^ CFU/μg (Figure 5B). The exception was *B. longum* subsp. *longum* NCIMB8809 which did not yield any colonies following transformation of pCL007 propagated in *E. coli* . However, propagation of the plasmid in an *E. coli* strain expressing the *B. longum* subsp. *longum* methylase B8809_607 (O'Callaghan et al. 2015) resulted in a transformation rate of 5.3 × 10^6^ cfu/μg of DNA indicating that the type II restriction enzyme B8809_606 was acting as an effective barrier to plasmid transfer (Table S6). gRNAs targeting the *upp* gene in each species were used to determine if CRISPRi could induce resistance to 5‐fluorouracil. All tested bifidobacterial species were shown to exhibit sensitivity to 5‐FU ranging from 0.1 μM to 0.5 μM 5‐FU. In all species tested the CRISPRi system targeting *upp* had increased resistance to 5‐FU with 1 × 10^3^–1 × 10^5^ fold higher colony counts observed in clones expressing CRISPRi with an *upp*‐targeting gRNA compared to parental WT strains (Figure 5C,D). The increase in resistance was significant in all cases except for *B. longum* subsp. *infantis* where there appears to be a large effect size, but it does not reach statistical significance (*p* = 0.156). Overall, this demonstrates efficient gene targeting across several species of *Bifidobacterium* without the requirement for extensive optimization of transformation.

### 3.5 Targeted Disruption of Carbohydrate Metabolism in *B. longum* Subsp*. Infantis*

**FIGURE 6:**
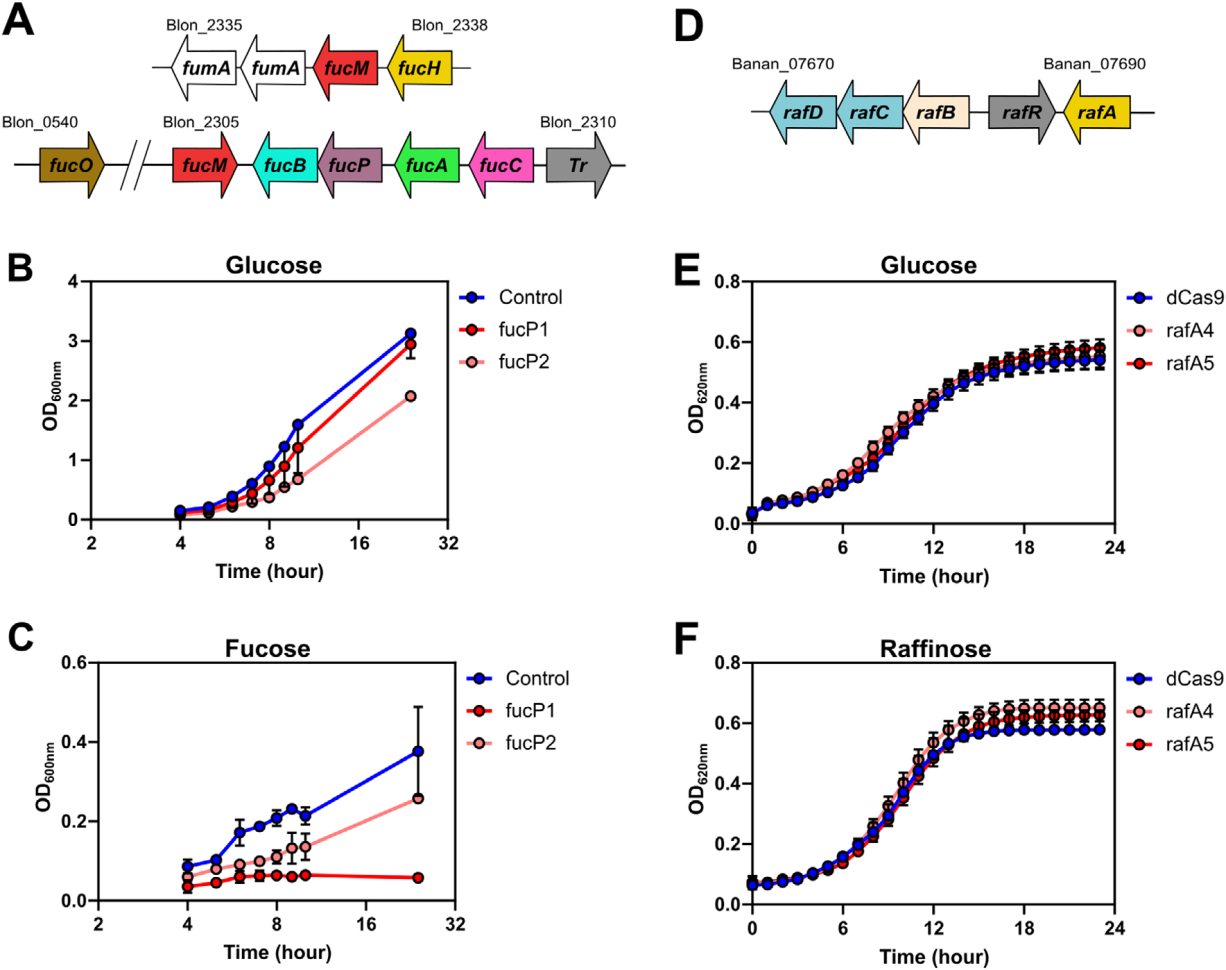
CRISPRi repression of fucose metabolism in *B. longum* subsp. *infantis* and *B. animalis* subsp. *animalis*. (A) Schematic of the operons associated with fucose metabolism in *B. longum* subsp. *infantis* ATCC 15697. (B) Growth of *B. longum* subsp. *infantis* ATCC 15697 CRISPRi strains with dCas9 alone or in combination with gRNAs targeting *fucP* in media containing glucose. *N* = 8 Error bars represent standard deviation (C) Growth of *B. infantis* ATCC 15697 and CRISPRi strains with dCas9 alone and in combination with gRNAs targeting *fucP* in media containing fucose. *N* = 8 Error bars represent standard deviation. (D) Schematic of raffinose operon in *B. animalis* subsp. *animalis* ATCC 25527. (E) Growth of *B. animalis* subsp. *animalis* ATCC 25527 CRISPRi strains with dCas9 alone or in combination with gRNAs targeting *rafA* in media containing glucose. *N* = 13 Error bars represent standard deviation (F) CRISPRi strains with dCas9 alone or in combination with gRNAs targeting *rafA* in media containing raffinose. *N* = 13 Error bars represent standard deviation.

Human milk contains a large proportion of non‐digestible human milk oligosaccharides (HMOs) (Ballard and Morrow 2013) which can participate in innate immunity by directly interfering with pathogen colonization (Newburg 2009) and indirectly by acting as a prebiotic to HMO‐utilising bacteria including several species of *Bifidobacterium* (Wang et al. 2015; di Gioia et al. 2014). Although there are over 200 structurally distinct HMOs (Sela and Mills 2010) most females primarily produce fucosylated HMOs with the fucosylated trisaccharide 2′‐fucosyllactose (2‐FL) being the most abundant (Asakuma et al. 2011; Kobata 2010). *B. longum* subsp. *infantis* ATCC 15697 is not only capable of metabolising 2‐FL but can also take up free fucose via a fucose permease and utilise it via a shared pathway with 2‐FL (Figure S3). A previously characterised insertion mutagenesis mutant of the fucose permease (*fucP*) in *B. longum* subsp. *infantis* ATCC 15697 demonstrated that this was essential for uptake and utilisation of fucose (Higgins and Ryan 2021). Two distinct gRNAs were designed to target the fucose permease *fucP* with one gRNA (fucP1) resulting in complete inhibition of growth when fucose was used as a sole carbon source (Figure 6C) (*p* < 0.001, repeated measures ANOVA), with minimal impact on growth in the presence of glucose (Figure 6B) (*p* = 0.06, repeated measures ANOVA) demonstrating specific targeting of the fucose utilisation pathway.

### 3.6 Targeted Disruption of Carbohydrate Metabolism in *B. animalis* Subsp*. Animalis*

*B. animalis* subsp. *animalis* ATCC 25527 is capable of growth in the presence of raffinose and contains a gene cluster (Figure 6D) orthologous to the raffinose cluster found in *B. breve* UCC2003 (Figure 4A). We used the one‐plasmid CRISPRi system to target the putative alpha‐galactosidase Banan_07690 responsible for raffinose metabolism (*rafA*) with 2 gRNAs and assessed the impact on *B. animalis* growth in the presence of different sugars. No impact on growth was observed in the presence of glucose (Figure 6E) or in the presence of raffinose with both *rafA* gRNAs (Figure 6F).

### 3.7 Targeted Disruption of Carbohydrate Metabolism in *B. longum* Subsp*. Longum*

**FIGURE 7:**
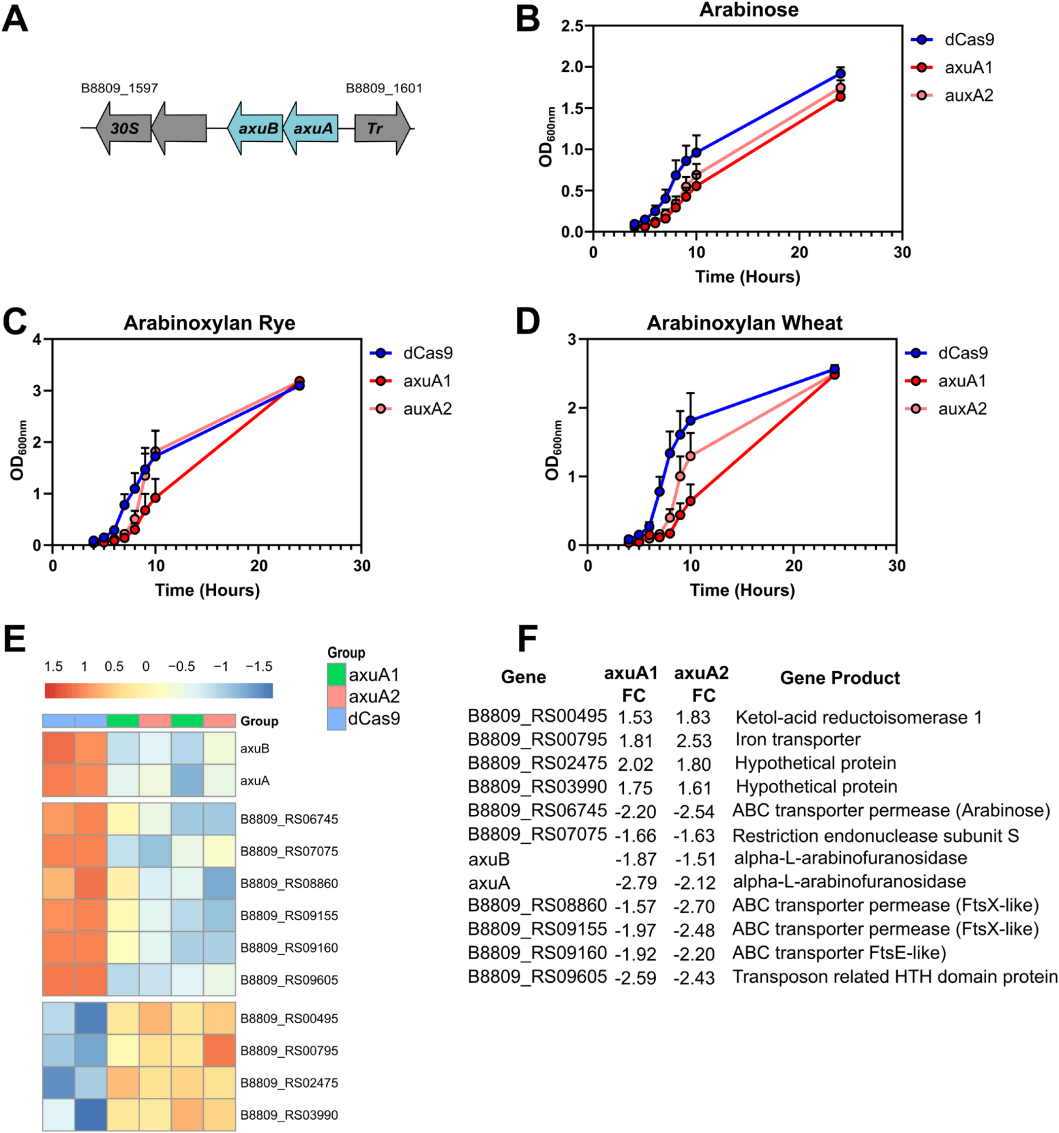
CRISPRi repression of carbohydrate metabolism in *B. longum* subsp. *longum*. (A) Schematic of *axu* gene cluster in *B. longum* subsp. *longum* NCIMB 8809. (B) Growth of *B. longum* subsp. *longum* NCIMB 8809 and CRISPRi strains with dCas9 alone or in combination with gRNAs targeting *axuA* in media containing arabinose. *N* = 8, error bars represent standard deviation. (C) Growth of *B. longum* subsp. *longum* NCIMB 8809 and CRISPRi strains with dCas9 alone or in combination with gRNAs targeting *axuA* in media containing arabinoxylan from rye. *N* = 8, error bars represent standard deviation. (D) Growth of *B. longum* subsp. *longum* NCIMB 8809 and CRISPRi strains with dCas9 alone or in combination with gRNAs targeting *axuA* in media containing arabinoxylan from wheat. *N* = 8, error bars represent standard deviation. (E) Heatmap of genes differentially expressed following CRISPRi targeting of *axuA*. (F) Top ranked genes differentially expressed in *B. longum* subsp. *longum* following CRISPRi targeting of *axuA*.

Recently two genes responsible for the degradation of arabinoxylan by certain *B. longum* subsp. *longum* strains have been described: *axuA* and *axuB* (Friess et al. 2024) (Figure 7A). Here we targeted the α‐arabinofuranosidase‐encoding gene *axuA* with 2 gRNAs. In both cases clones transformed with CRISPRi targeting *axuA* had a slight reduction in the growth rate in the presence of arabinose compared to *B. longum* transformed with *sth1* dCas9 alone although this did not reach significance (axuA1—*p* = 0.055; axuA2—*p* = 0.21, repeated measures ANOVA) (Figure 7B). The axuA1 gRNA inhibited growth in the presence of arabinoxylan isolated from rye (Figure 7C) or wheat (Figure 7D) resulting in an increased lag time; although this was only significant in the case of arabinoxylan from wheat (*p* < 0.001) and not with arabinoxylan from rye (*p* = 0.18). The axuA2 gRNA did not significantly reduce growth in the presence of arabinoxylan from rye (*p* = 1) or wheat (*p* = 0.06). In all cases CRISPRi strains were able to overcome growth inhibition to reach a final OD equivalent to the dCas9‐only strain. Similar to the delayed growth profiles observed here, previous reports have shown that when an essential gene is repressed in bacteria, loss‐of‐function mutations arise in the CRISPRi system which results in recovery of bacterial growth after an extended lag phase (de Bakker et al. 2022; Liu et al. 2021). To determine the effect of CRISPRi at the transcriptional level, an RNA‐seq analysis was carried out on *B. longum* subsp. *longum* strains transformed with dCas9 alone or in combination with the *axuA* targeting gRNAs and grown on arabinose. In total 12 genes were differentially expressed across both *axuA* gRNAs with 8 genes downregulated and 4 genes upregulated (Figure 7E and Table S7). The top differentially expressed genes include the target gene *axuA* and *axuB* which is in the same operon (Figure 7E,F). The other differentially expressed genes do not contain any predicted off‐target sites for either gRNA and the strong overlap in differentially expressed genes with two different gRNAs suggests that changes in the expression of these non‐target genes are more likely an adaptation of these strains to the loss of *axuA*/*axuB* expression.

## 4 Conclusion

Bifidobacteria are early colonisers of and persistent residents in the intestinal tract of humans and other animals and are widely reported to promote human health. However, their refractory nature to genetic manipulation has hampered functional genomic studies and genetic engineering applications for the benefit of human physiology and pathology. To overcome this, we developed a CRISPRi platform for rapid programmable gene repression in bifidobacteria. Initial testing of two orthologs of dCas9 from *S. pyogenes* and *S. thermophilus* found that the *Spy* dCas9 was nonfunctional in *B. breve* (Figure 1) a finding in line with a previous study in mycobacteria showing that Spy dCas9 may not be an optimal choice of CRISPR system (Rock et al. 2017). In contrast, the *Sth1* dCas9 system was capable of effectively suppressing gene expression of the nanoluciferase reporter gene and was easily reprogrammed to suppress expression of endogenous genes (*upp*, *Bbr_0430*, *rafA*, *rbsA*) in *B. breve* . The superior performance of *Sth1* dCas9 in bifidobacteria may stem from the use of a lower GC PAM sequence, a lower PAM density within the host genome, or the use of longer gRNA sequences, although it must be noted that in mycobacteria expression of Spy dCas9 resulted in proteotoxic stress (Rock et al. 2017).

To apply this CRISPRi system to other species of bifidobacteria we developed a single‐plasmid system based on the *B. catenulatum* pBC1 replicon which is capable of replicating in a range of bifidobacterial species (Álvarez‐Martín et al. 2008). This system is readily reprogrammed by inserting a unique 22 bp targeting region into the gRNA expression cassette. Using this system we successfully targeted genes involved in nucleotide metabolism (Figure 5) and carbohydrate metabolism (Figures 6 and 7) in *B. breve* , *B. animalis* subsp. *animalis*, *B. longum* subsp. *longum*, *B. longum* subsp. *infantis*, and *B. pseudocatenulatum* demonstrating the utility of this system across three distinct phylogenetic groups of bifidobacteria (Brandt and Barrangou 2016).

Of the 17 gRNAs targeting the non‐template strand, 13 were effective in repressing gene expression and in each case, all colonies selected had the expected gain or loss of function phenotype demonstrating the efficiency of this CRISPRi system for targeted gene knockdown. In contrast to the widely used *Spy* dCas9 system, the *Sth1* dCas9 system has a longer PAM sequence which limits its ability to target a specific DNA sequence. However, sequences beyond the canonical PAM sequence are tolerated by *Sth1* dCas9 which mitigates this apparent limitation (Rock et al. 2017). When these relaxed parameters are applied to the genome of *B. breve* UCC2003 we could bioinformatically design *Sth1* gRNAs approximately every 86 base pairs, with a theoretical library capable of targeting 1793 out of 1824 genes not accounting for repetitive sequences such as transposons (Table S5).

Unlike current methods such as insertional mutagenesis, the CRISPRi system described herein provides a robust platform for further development of gene editing tools in bifidobacteria including multiplexing which facilitates targeting of redundant genes and pathways (Jiang et al. 2013, 2015; Huang et al. 2015; Cobb et al. 2014), tunable levels of gene repression through the incorporation of inducible expression systems for dCas9 (Qi et al. 2013) and the gRNA or through lowering the gRNA‐target site complementarity (Larson et al. 2013) (Figure 2), and genome‐wide scalability for functional genomic screens (Qi et al. 2013; Rock et al. 2017; Spoto et al. 2022; Liu et al. 2024). Tuneable levels of gene repression would also permit the targeting of the 453 essential genes identified in *B. breve* UCC2003 (Ruiz et al. 2017) enabling the investigation of the fitness of these genes under different environmental conditions.

## Author Contributions

C.L. and D.S. proposed and led this study. C.L. and L.F. collected the data and performed the analysis. C.L. and L.F. drafted the original manuscript. L.F., D.S., and C.L. reviewed and edited the manuscript. All authors have reviewed and approved it for publication.

## Conflicts of Interest

The authors declare no conflicts of interest.

## Supporting information


**Figure S1:** Plasmid map of pCL002.
**Figure S2:** Plasmid map of pCL007.
**Figure S3:** Fucose metabolism pathway in *B. infantis* ATCC15697.
**Table S1:** List of bacteria strains used in this study.
**Table S2:** Primer sequences used in this study. Sequences in bold represent restriction sites used for cloning.
**Table S3:** List of plasmids used in this study.
**Table S4:** gRNA target sequences used in this study.
**Table S6:** Restriction‐Modification systems in target Bifidobacteria strains.

**Table S5:** Putative Sth1 gRNA target sites in the *B. breve* UCC2003 genome.

**Table S7:** Differential gene expression of CRISPRi in *B. longum* NCIMB8809.

## Data Availability

The data that support the findings of this study are available from the corresponding author upon reasonable request.
