# Supplementary material for: A CRISPRi Gene Regulation System for Bifidobacteria
Source: Microb Biotechnol. 2025 Nov 6;18(11):e70260. doi: 10.1111/1751-7915.70260 (PMC12592239; doi:10.1111/1751-7915.70260)
Supplement: Supplementary file 1 — Figure S1: Plasmid map of pCL002. Figure S2: Plasmid map of pCL007. Figure S3: Fucose metabolism pathway in B. infantis ATCC15697. Table S1: List of bacteria strains used in this study. Table S2: Primer sequences used in this study. Sequences in bold represent restriction sites used for cloning. Table S3: List of plasmids used in this study. Table S4: gRNA target sequences used in this study. Table S6: Restriction‐Modification systems in target Bifidobacteria strains. [file MBT2-18-e70260-s003.docx]

**Supplementary Figure S1:** Plasmid map of pCL002

**
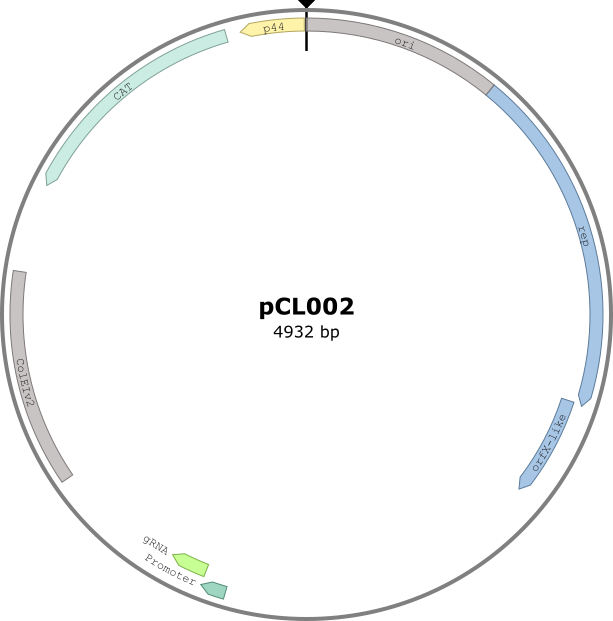
**

**LOCUS pCL002 4932 bp ds-DNA circular 27-AUG-2025**

**DEFINITION .**

**FEATURES Location/Qualifiers**

**misc_feature 1..538**

**/label="ori"**

**misc_feature 539..1486**

**/label="rep"**

**misc_feature 1483..1773**

**/label="orfX-like"**

**misc_feature 2690..2759**

**/label="Promoter"**

**misc_feature 2760..2865**

**/label="gRNA"**

**Origin 3228..3816**

**/label="ColEIv2"**

**misc_feature complement(4062..4712)**

**/label="CAT"**

**Promoter complement(4752..4926)**

**/label="p44"**

**ORIGIN**

**1 ccagagtaac accacggtca cacaagtgga acaccgttgt ggcacaagcc gcgcgagcgg**

**61 tctggaggct ccgccgacca tggggtacgg gtcttcccgg caagccgatt cgcaccgcga**

**121 atcgagtgat tgtagctaca atcacgtcgc ttgctactcc ggccttgcgg ctttttcctc**

**181 ggcctcgggt tgttgctcct gcgccgaagg gcgcaggagg tgctgcgttc ggttcatgta**

**241 catgaaccgt ctagcttgct taccttcgat ttgatggaca tctccatgtg gatgtccatg**

**301 gacatatcca tgtgggtgtc catagacgtg tccatgtggg tatccatgga tatatccatc**

**361 tgagcgcata ggaagcgcat gagggtggat gatcgttcgg tttggggtgt ttttggccag**

**421 atttcgacag ttttgggtaa ctctgggaag gagcagccca tgctttgctg ggctgaaata**

**481 tctgacttgg ttttaggcga acttgaccag tggtcaagta cgacgtacac tagtgcccat**

**541 gtctgacgag tattcgcagc cgacgcttga gctgtcgcgc acgttcgagg gctggtggct**

**601 gccccggcgt ccgctgtgct gcgacgacga ctacagccag ctggtgcgcc ggagccgcac**

**661 cgacgcgctc agatgcaagc acatcgaggc gaatccctcg gcgctggtga acacgatcgt**

**721 ggtggacatc gacgacgcga acgccaaggc gatggccctg tgggggcacc gtgggatgct**

**781 gccgaactgg atcgcggaga acccggccaa cgggcacgct cacgcgggct gggtgctgac**

**841 ctaccccgtg ccccgcaccg acatggcccg gctcaaaccg ctgaagctcc tgcacgccgt**

**901 caccgagggg ctgcgccgca gcgtggacgg cgacgagggc tattccggcc tgctgatgaa**

**961 gaacccgctg agcgacgcgt gggacagcga cctgtgccgc gaggacacct acgacctgcc**

**1021 cgacctcgtg gccgcgctgg aggagcacgg ggacatgccg cccaagagct ggacgcgcac**

**1081 caaacgcgcc cgcgaggtcg gcgtgggccg caactgcacc ttgttcgacg aggcccgcac**

**1141 cctcgcctac cgtcaggtgc gccgactgcc cgaccgcacg cccgcctcct ccgacctgct**

**1201 gcgcgagtac gtgcgccgca cctgccacga aatcaacgcc tcgttccccg acccgctgcc**

**1261 cgtgcgcgag gtcaacgaca ccgccaagag catccacaag tggatcacca cgcgaagccg**

**1321 catgtggagg gacggtgccg tcgccaacgc cgccacgttc gtcgccatcc aatccgcgcg**

**1381 cggacgcaaa ggcggtcgcg gaaacaaacg cgacaagcaa gggaacgttc aaaatgcctt**

**1441 caagcaaaag gcagagctgt tcggcaagga gatgatgggg caatgacgat tcagacgatt**

**1501 cgcaagaagc gtccgcttcc cgccaaggag ctggcggcaa tgtacgacgt ctccgtgcgc**

**1561 actattcagc gatgggcttc acaaacccgt aaggactgga tagacgaaca ggcaacgttg**

**1621 cgcgaatcca tccgtgccta ccacgatgac gagggccata cgtggccgca gaccgccgag**

**1681 cacttcggca tgagccaaga cgcggtgcgt agccgctgct accgcgcgcg caaggagcgt**

**1741 gcggccgagg caaaagccgc aaggccggag tagcaagtga cgtgaacgta tatacgttca**

**1801 ctcgattcgc accgcgaatc ggcttgccgg gaagacccgt accccatggt cggctgcgcc**

**1861 tccagaccgc tcgcgcggca tggtgacacc gcggtgtcac ctatgggaac acccgtgtgc**

**1921 cacgaatgat ggcacacggg tgttccgtct tcgcgcggcg gcggtcagac ggctccggcg**

**1981 agctgccggc gcacgtactc gctgcggctg atgtggtagc gcccggcggt ctccgtgacc**

**2041 ttctccagct gactcttggg cagcttgacg gtcatgctga ccatctcctc gccgtgctcg**

**2101 gggagcatgt gcagcgcgta gtggacgggt ccggttatgc cgtggcccgc ggtctcggac**

**2161 tccatgcgct ccacgtcgtc cctgacctga tcctcgccca tgccgaacat cgccaacagc**

**2221 tccctgtcgc gttcgctgaa atcgctcatc ggtcgctcct tcccccgcgc aggcgggcta**

**2281 tctcgcttcg ggtcttcttc gtcggcggcg tcatggcgtg gtagatgagc gcgcggtcgc**

**2341 cgcgctgctc gtagaccatc tccacgtccc tgccgcgccc gtcgaggccg atgcagaccc**

**2401 acgtgccgtt gtccctgcgg gcgtccacca tgacgctcct gaacgccgca agcacgtcct**

**2461 ccacggacag ctcgggatgc cgctcatgca cgcgcggcag cacgtagacc ctctttaatt**

**2521 tggttatatg aattttgctt attaacgatt cattataacc acttattttt tgtttggttg**

**2581 ataatgaact gtgctgatta caaaaatact aaaaatgccc atattttttc ctccttataa**

**2641 aattagtata attatagcac gcccgcatgc catggtaccc ggggaattct ctgaccaggg**

**2701 aaaatagccc tctgacctgg ggatttgcga tgcgtgtgct cggttgatat aatctgggag**

**2761 agaagagcga acaggctctt ctgtttttgt actcgaaaga agctacaaag ataaggcttc**

**2821 atgccgaaat caacaccctg tcattttatg gcagggtgtt tttttccaat tattgaaggc**

**2881 ctccctaacg gggggccttt ttttgtttct ggtctccctc tagaactagc gattctgaaa**

**2941 tcaccattta aaaaactcca atcaaataat tttataaagt tagtgtatca ctttgtaatc**

**3001 ataaaaacaa caataaagct acttaaatat agatttataa aaaacgttgg cgaaaacgtt**

**3061 ggcgattcgt tggcgattga aaaacccctt aaacccttga gccagttggg atagagcgta**

**3121 gcaattgacg gttatccaca gaatcagggg ataacgcagg aaagaacatg tgagcaaaag**

**3181 gccagcaaaa cgcgaggaac cgtaaaaagg ccgcgttgct ggcgttcttg agatcctttt**

**3241 tttctgcgcg taatctgctg cttgcaaaca aaaaaaccac cgctaccagc ggtggtttgt**

**3301 ttgccggatc aagagctacc aactcttttt ccgaaggtaa ctggcttcag cagagcgcag**

**3361 ataccaaata ctgttcttct agtgtagccg tagttaggcc accacttcaa gaactctgta**

**3421 gcaccgccta catacctcgc tctgctaatc ctgttaccag tggctgctgc cagtggcgat**

**3481 aagtcgtgtc ttaccgggtt ggactcaaga cgatagttac cggataaggc gcagcggtcg**

**3541 ggctgaacgg ggggttcgtg cacacagccc agcttggagc gaacgaccta caccgaactg**

**3601 agatacctac agcgtgagct atgagaaagc gccacgcttc ccgaagggag aaaggcggac**

**3661 aggtatccgg taagcggcag ggtcggaaca ggagagcgca cgagggagct tccaggggga**

**3721 aacgcctggt atctttatag tcctgtcggg tttcgccacc tctgacttga gcgtctattt**

**3781 ttgtgatgct cgtcaggggg gcggagccta tggaaaaacg ccagcaacgc ggcctttttt**

**3841 agcgattctg aaatcaccat ttaaaaaact ccaatcaaat aattttataa agttagtgta**

**3901 tcactttgta atcataaaaa caacaataaa gctactagtg acacgaaaaa caagttaagg**

**3961 gatgcagttt atgcatccct taacaacgca agaagccccg accggttggg gtcggggctt**

**4021 ctgtttttag tactactggt ggatttcggt tttggtgggg attacaggag ccagtcgttc**

**4081 gggcggtccg acagttcttg aatggagttc atgaacaggc cggcgtggta gccgtcgcac**

**4141 acggagtggt gcacttgtaa cgacagcggc aggtagatgg agttgccctt gttgatgaac**

**4201 ttgccggcgg tgataatcgg cagcaggtag ttggagttgt tgttgatgtt caggttgaag**

**4261 ccggtgaagg aggtccacgg gataatcgag agggagaagg cgttttccgg aatcggggtc**

**4321 ttcgggaaca gcttgccgga gccgttgtac ttctccacgt cggagaggta caggtcgtag**

**4381 aactccttga agtcgttctt caccggggtc cagatgccgg agaaggtctt ggacacgccg**

**4441 tcgaagatgg tgtacagcgg ctccagcttg tcccagtagc ccagttcgcc gtcggagttg**

**4501 tagccggtgc ggaaggcggt gttggagtta atcacgcggg taactaaaaa gatgaaggcc**

**4561 gggtagaact tgtagccctc ctgcttgatg ttgcggtaca gcacggagat atcaatctcg**

**4621 gtggtgatgg agaaggtggt ctgctggttg aggtagtggt taaaaatttc cttgcgcttc**

**4681 cagttatcta agtcaatctt gttgaagttc ataaggtgtg ctcctttccc tcacacatca**

**4741 cgattcatat ttttttcaat aaattaggta agtgtacaga taattttata tacaattaca**

**4801 ctcattaatt attttaacat aaaatcacag taaaacaaga gtttacctgt gattttttca**

**4861 gttttttatt aaattattct ctactttgtc tacttattga aaaaattgct atcgtcccgt**

**4921 ctcctactta ta**

**//**

**Supplementary Figure S2:** Plasmid map of pCL007

**
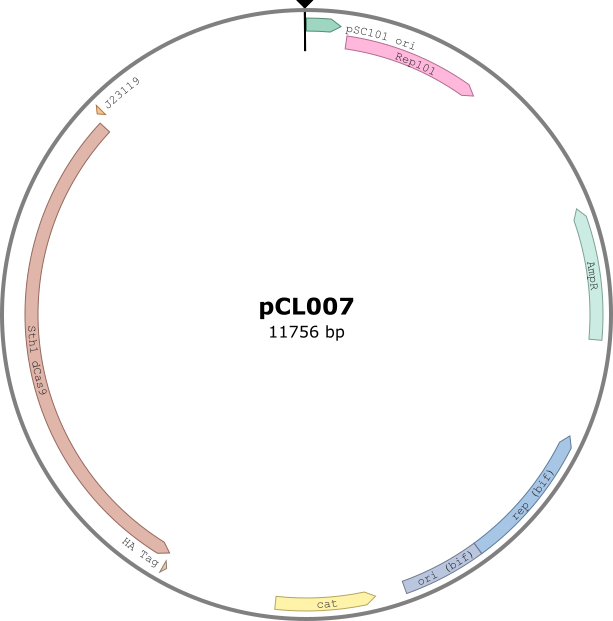
**

**LOCUS pCL007 11756 bp ds-DNA circular 27-AUG-2025**

**DEFINITION .**

**FEATURES Location/Qualifiers**

**misc_feature 1..223**

**/label="pSC101 ori"**

**misc_feature 271..1221**

**/label="Rep101"**

**misc_feature complement(2242..3102)**

**/label="AmpR"**

**misc_feature complement(3748..4695)**

**/label="rep (bif)"**

**misc_feature 4696..5233**

**/label="ori (bif)"**

**misc_feature complement(5429..6079)**

**/label="cat"**

**misc_feature complement(6821..6847)**

**/label="HA Tag"**

**misc_feature complement(6854..10216)**

**/label="Sth1 dCas9"**

**misc_feature complement(10248..10282)**

**/label="J23119"**

**ORIGIN**

**1 gagttataca cagggctggg atctattctt tttatctttt tttattcttt ctttattcta**

**61 taaattataa ccacttgaat ataaacaaaa aaaacacaca aaggtctagc ggaatttaca**

**121 gagggtctag cagaatttac aagttttcca gcaaaggtct agcagaattt acagataccc**

**181 acaactcaaa ggaaaaggac tagtaattat cattgactag cccatctcaa ttggtatagt**

**241 gattaaaatc acctagacca attgagatgt atgtctgaat tagttgtttt caaagcaaat**

**301 gaactagcga ttagtcgcta tgacttaacg gagcatgaaa ccaagctaat tttatgctgt**

**361 gtggcactac tcaaccccac gattgaaaac cctacaagga aagaacggac ggtatcgttc**

**421 acttataacc aatacgctca gatgatgaac atcagtaggg aaaatgctta tggtgtatta**

**481 gctaaagcaa ccagagagct gatgacgaga actgtggaaa tcaggaatcc tttggttaaa**

**541 ggctttgaga ttttccagtg gacaaactat gccaagttct caagcgaaaa attagaatta**

**601 gtttttagtg aagagatatt gccttatctt ttccagttaa aaaaattcat aaaatataat**

**661 ctggaacatg ttaagtcttt tgaaaacaaa tactctatga ggatttatga gtggttatta**

**721 aaagaactaa cacaaaagaa aactcacaag gcaaatatag agattagcct tgatgaattt**

**781 aagttcatgt taatgcttga aaataactac catgagttta aaaggcttaa ccaatgggtt**

**841 ttgaaaccaa taagtaaaga tttaaacact tacagcaata tgaaattggt ggttgataag**

**901 cgaggccgcc cgactgatac gttgattttc caagttgaac tagatagaca aatggatctc**

**961 gtaaccgaac ttgagaacaa ccagataaaa atgaatggtg acaaaatacc aacaaccatt**

**1021 acatcagatt cctacctaca taacggacta agaaaaacac tacacgatgc tttaactgca**

**1081 aaaattcagc tcaccagttt tgaggcaaaa tttttgagtg acatgcaaag taagtatgat**

**1141 ctcaatggtt cgttctcatg gctcacgcaa aaacaacgaa ccacactaga gaacatactg**

**1201 gctaaatacg gaaggatctg aggttcttat ggctcttgta tctatcagtg aagcatcaag**

**1261 actaacaaac aaaagtagaa caactgttca ccgttacata tcaaagggaa aactgtccat**

**1321 atgcacagat gaaaacggtg taaaaaagat agatacatca gagcttttac gagtttttgg**

**1381 tgcattcaaa gctgttcacc atgaacagat cgacaatgta acagatgaac agcatgtaac**

**1441 acctaataga acaggtgaaa ccagtaaaac aaagcaacta gaacatgaaa ttgaacacct**

**1501 gagacaactt gttacagctc aacagtcaca catagacagc ctgaaacagg cgatgctgct**

**1561 tatcgaatca aagctgccga caacacggga gccagtgacg cctcccgtgg ggaaaaaatc**

**1621 atggcaattc tggaagaaat agcgctttca gccggcaaac cggctgaagc cggatctgcg**

**1681 attctgataa caaactagca acaccagaac agcccgtttg cgggcagcaa aacccgtact**

**1741 tttggacgtt ccggcggttt tttgtggcga gtggtgttcg ggcggtgcgc gcaagatcca**

**1801 ttatgttaaa cgggcgagtt tacatctcaa aaccgcccgc ttaacaccat cagaaatcct**

**1861 cagcgcgatt ttaagcacca accccccccc gtaacaccca aatccatact gaaagtggct**

**1921 ttgttgaata aatcagattt cgggtaagtc tcccccgtag cgggttgtgt tttcaggcaa**

**1981 tacgcacgct ttcaggcata cctgctttcg tcattttgtt cagcgctcgt accagggcca**

**2041 tagcctccgc aacctgacca tcgtagtcac gcagcgtcag tgaacccccg aacagagatc**

**2101 tgacgctcag tggaacgaaa actcacgtta agggattttg gtcatgagat tatcaaaaag**

**2161 gatcttcacc tagatccttt taaattaaaa atgaagtttt aaatcaatct aaagtatata**

**2221 tgagtaaact tggtctgaca gttaccaatg cttaatcagt gaggcaccta tctcagcgat**

**2281 ctgtctattt cgttcatcca tagttgcctg actccccgtc gtgtagataa ctacgatacg**

**2341 ggagggctta ccatctggcc ccagtgctgc aatgataccg cgagacccac gctcaccggc**

**2401 tccagattta tcagcaataa accagccagc cggaagggcc gagcgcagaa gtggtcctgc**

**2461 aactttatcc gcctccatcc agtctattaa ttgttgccgg gaagctagag taagtagttc**

**2521 gccagttaat agtttgcgca acgttgttgc cattgctaca ggcatcgtgg tgtcacgctc**

**2581 gtcgtttggt atggcttcat tcagctccgg ttcccagcga tcaaggcgag ttacatgatc**

**2641 ccccatgttg tgcaaaaaag cggttagctc cttcggtcct ccgatcgttg tcagaagtaa**

**2701 gttggccgca gtgttatcac tcatggttat ggcagcactg cataattctc ttactgtcat**

**2761 gccatccgta agatgctttt ctgtgactgg tgagtactca accaagtcat tctgagaata**

**2821 gtgtatgcgg cgaccgagtt gctcttgccc ggcgtcaata cgggataata ccgcgccaca**

**2881 tagcagaact ttaaaagtgc tcatcattgg aaaacgttct tcggggcgaa aactctcaag**

**2941 gatcttaccg ctgttgagat ccagttcgat gtaacccact cgtgcaccca actgatcttc**

**3001 agcatctttt actttcacca gcgtttctgg gtgagcaaaa acaggaaggc aaaatgccgc**

**3061 aaaaaaggga ataagggcga cacggaaatg ttgaatactc atactcttcc tttttcaata**

**3121 ttattgaagc atttatcagg gttattgtct catgagcgga tacatatttg aatgtattta**

**3181 gaaaaataaa caaatagggg ttccgcgcac atttccccga aaagtgccac ctaaattgta**

**3241 agcgttgcat gctcgccgga gccgtctgac cgccgccgcg cgaagacgga acacccgtgt**

**3301 gccatcattc gtggcacacg ggtgttccca taggtgacac cgcggtgtca ccatgccgcg**

**3361 cgagcggtct ggaggcgcag ccgaccatgg ggtacgggtc ttcccggcaa gccgattcgc**

**3421 ggtgcgaatc gagtgaacgt atatacgttc acgtcacttg ctactccggc cttgcggctt**

**3481 ttgcctcggc cgcacgctcc ttgcgcgcgc ggtagcagcg gctacgcacc gcgtcttggc**

**3541 tcatgccgaa gtgctcggcg gtctgcggcc acgtatggcc ctcgtcatcg tggtaggcac**

**3601 ggatggattc gcgcaacgtt gcctgttcgt ctatccagtc cttacgggtt tgtgaagccc**

**3661 atcgctgaat agtgcgcacg gagacgtcgt acattgccgc cagctccttg gcgggaagcg**

**3721 gacgcttctt gcgaatcgtc tgaatcgtca ttgccccatc atctccttgc cgaacagctc**

**3781 tgccttttgc ttgaaggcat tttgaacgtt cccttgcttg tcgcgtttgt ttccgcgacc**

**3841 gcctttgcgt ccgcgcgcgg attggatggc gacgaacgtg gcggcgttgg cgactgcacc**

**3901 gtccctccac atgcggcttc gcgtggtgat ccacttgtgg atgctcttgg cggtgtcgtt**

**3961 gacctcgcgc acgggcagcg ggtcggggaa cgaggcgttg atttcgtggc aggtgcggcg**

**4021 cacgtactcg cgcagcaggt cggaggaggc gggcgtgcgg tcgggcagtc ggcgcacctg**

**4081 acggtaggcg agggtgcggg cctcgtcgaa caaggtgcag ttgcggccca cgccgacctc**

**4141 gcgggcgcgt ttggtgcgcg tccagctctt gggcggcatg tccccgtgct cttcgagcgc**

**4201 ggccacgagg tcgggcaggt cgtaggtgtc ctcgcggcac aggtcgctgt cccacgcgtc**

**4261 gctcagcggg ttcttcatca gcaggccgga atagccctcg tcgccgtcca cgctgcggcg**

**4321 cagcccctcg gtgacggcgt gcaggagctt cagcggtttg agccgggcca tgtcggtgcg**

**4381 gggcacgggg taggtcagca cccagcccgc gtgagcgtgc ccgttggccg ggttctccgc**

**4441 gatccagttc ggcagcatcc cacggtgccc ccacagggcc atcgccttgg cgttcgcgtc**

**4501 gtcgatgtcc accacgatcg tgttcaccag cgccgaggga ttcgcctcga tgtgcttgca**

**4561 tctgagcgcg tcggtgcggc tccggcgcac cagctggctg tagtcgtcgt cgcagcacag**

**4621 cggacgccgg ggcagccacc agccctcgaa cgtgcgcgac agctcaagcg tcggctgcga**

**4681 atactcgtca gacatgggca ctagtgtacg tcgtacttga ccactggtca agttcgccta**

**4741 aaaccaagtc agatatttca gcccagcaaa gcatgggctg ctccttccca gagttaccca**

**4801 aaactgtcga aatctggcca aaaacacccc aaaccgaacg atcatccacc ctcatgcgct**

**4861 tcctatgcgc tcagatggat atatccatgg atacccacat ggacacgtct atggacaccc**

**4921 acatggatat gtccatggac atccacatgg agatgtccat caaatcgaag gtaagcaagc**

**4981 tagacggttc atgtacatga accgaacgca gcacctcctg cgcccttcgg cgcaggagca**

**5041 acaacccgag gccgaggaaa aagccgcaag gccggagtag caagcgacgt gattgtagct**

**5101 acaatcactc gattcgcggt gcgaatcggc ttgccgggaa gacccgtacc ccatggtcgg**

**5161 cggagcctcc agaccgctcg cgcggcttgt gccacaacgg tgttccactt gtgtgaccgt**

**5221 ggtgttactc tgggcatgcg attgttaagg gttccgaggc tcaacgtcaa taaagcaatt**

**5281 ggaataaaga agcgaaaaag gagaagtcgg ttcagaaaaa gaaggatatg gatctggagc**

**5341 tgtaatataa aaaccttctt caactaacgg ggcaggttag tgacattaga aaaccgactg**

**5401 taaaaagtac agtcggcatt atctcatatt ataaaagcca gtcattaggc ctatctgaca**

**5461 attcctgaat agagttcata aacaatcctg catgataacc atcacaaaca gaatgatgta**

**5521 cctgtaaaga tagcggtaaa tatattgaat tacctttatt aatgaatttt cctgctgtaa**

**5581 taatgggtag aaggtaatta ctattattat tgatatttaa gttaaaccca gtaaatgaag**

**5641 tccatggaat aatagaaaga gaaaaagcat tttcaggtat aggtgttttg ggaaacaatt**

**5701 tccccgaacc attatatttc tctacatcag aaaggtataa atcataaaac tctttgaagt**

**5761 cattctttac aggagtccaa ataccagaga atgttttaga tacaccatca aaaattgtat**

**5821 aaagtggctc taacttatcc caataaccta actctccgtc gctattgtaa ccagttctaa**

**5881 aagctgtatt tgagtttatc acccttgtca ctaagaaaat aaatgcaggg taaaatttat**

**5941 atccttcttg ttttatgttt cggtataaaa cactaatatc aatttctgtg gttatactaa**

**6001 aagtcgtttg ttggttcaaa taatgattaa atatctcttt tctcttccaa ttgtctaaat**

**6061 caattttatt aaagttcatt tgatatgcct cctaaatttt tatctaaagt gaatttagga**

**6121 ggcttacttg tctgctttct tcattagaat caatcctttt ttaaaagtca atattactgt**

**6181 aacataaata tatattttaa aaatatccca ctttatccaa ttttcgtttg ttgaactaat**

**6241 gggtgcttta gttatggaaa agtatactga gaaaaaacaa agaaatcaag tatttcagaa**

**6301 atttattaaa cgtcatattg gagagaatca aatggattta gttgaagatt gcaatacatt**

**6361 tctgtctttt gtagctgata catgggctag ggcgctggca agtgtagcgg tcacgctgcg**

**6421 cgtaaccacc acacccgccg cgcttaatgc gccgctacag ggcgcgtccc attcgccatt**

**6481 caggctgcgc aactgttggg aagggcgatc ggtgcgggcc tcttcgctat tacgccagct**

**6541 ggcgaaaggg ggatgtgctg caaggcgatt aagttgggta acgccagggt tttcccagtc**

**6601 acgacgttgt aaaacgacgg ccagtgagcg cgcgtaatac gactcactat agggcgaatt**

**6661 gggtaccggg ccccccctcg aggtcgacgg tatcgataag cttgatatcg aattcctgca**

**6721 gcccggggga tccaagctgg ttcaagccta cgcgacgtca gggagaccag aaacaaaaaa**

**6781 aggccccccg ttagggaggc cttcaataat tggctattta agcgtagtcc ggcacgtcgt**

**6841 acgggtagga gccgaagtcc agcttcggct tgtcgccctc gttcttgatg atgtgctggt**

**6901 tgccgagcac gtcggtacgc accttgtaga tggagatgtt ggacttacct aaacccttct**

**6961 tgcactggcc ggaattagca acgttgccca gcaccttaat taaggcttcg ccgccctcga**

**7021 acttctgctt gtcgtacggc ttcagctcca cgtagtgctt ctgcttcggc atggtgcgcg**

**7081 acaggaagcg gaacagctgc tgctccttgg tctcggtgtc cttcacgagg agcaggtcgt**

**7141 tcttgtacag ggtaaacttg aactcggagt cggagtccac gccctccttc ttcttgatgt**

**7201 cgttgtactt ttcttgagaa attttgtagg tgccggtgcc cttgtcgaat tgtaagtcgg**

**7261 cgtacttcag gcccagaatt tcgtacttgc cggtggtctt gttgaagtac acgtcggcgc**

**7321 gccacgggga cacggattgt aacaccacct tgttgttgga gtccttcggg gtgatatcaa**

**7381 tgtggttgcc cagcttggaa tcgtagtact tcagggactt aatttccggg ccgttgccct**

**7441 tcttggagta cttgcggatg tagccgtgct cctccttgta cttgaggaac gggttgcacg**

**7501 ggacctcctt gcccttgtcg ttaatttgct tgttcgggta gttctccaga ataggctcaa**

**7561 taaccttctc gaaggtctgc gggtcgtggc ggtacatcag gaacttggac ttgtccttct**

**7621 tgtaaatttt catgaaggcg tcgtagccat cttgagtgta gatgtcctta attttgccca**

**7681 gcacgtaggt ctcgtcggcc ttgtccttgc ccaccttagc ttgacgggtg gcgtagatgg**

**7741 tggcgtcgga aattttgcgg ttgaacttgg agtcaacttg ataggagaac aggatggagt**

**7801 cttcgaactc cttggacttc agggtgtcaa cgaagtgctg gtacggggcc ttgaacacgg**

**7861 attccttgta ctcgtcgtcg gaaattaatt cgccggtctc gatatcgagg agctggtctt**

**7921 cggagtagga aactaaagtg ttcttctgct tcttccacag gttcagctgg gaggaggcgg**

**7981 cgataattaa ggcgtcaacg gcgtggtggt ggtaggtgtc gcgggtcttc tcgatgcccc**

**8041 agtggcggcg cagctgggag gtgaactggc cgcgcaccac ggacaccttg gtgtcaattt**

**8101 tgtgggcgcg gaagtgctct tgtaaggcgt tcagcaccac gcgcgaggcg tagcgggtgt**

**8161 caactaaatt gcgctcgatg aacttcttgc gcacgtcgaa cttggagatg tcttcctcgg**

**8221 tcagcaggta ctccttcttc ttgttcgaca gggtcttgga ctcgcgcacg aaggccttca**

**8281 gctcgcggaa ggaccaagcg tcgtccatcg aatctaaagc ttgatacggg gtgcgctggc**

**8341 ccttttcttg attggcggtg gcgtacacca gcaccttgtt agctaaagag tcgtcgaagg**

**8401 taatcgagag cggcaggatg gcgtccacct cgaactgatt ggagttgtta attaagtcgt**

**8461 ggatggagat ggttttgccg gtgtacaggc agcgctcacc ctgctggtgc cacaggcgaa**

**8521 ttttagtagc taactgcttg tggccgtgga acacggagtg cggcagctcg gccttgccgt**

**8581 tgtactggtt ggcggccttc agcatggcgg cgtccttctc gtccttgttg gccttctgaa**

**8641 ttttctggat ggccttcttc tcgtcgtctt cgttggtctc gcgggccatc tcaataacga**

**8701 tgttgtcgaa gtcgccgtac tccttgatgg cggcgttgac aattttgata gcttgacgca**

**8761 cggatttagc aaccaccggg ttgtaaattt cctcggtcag cagcttctca tcaatatact**

**8821 tggtcttgtt ggaggaggag gtggtcttct gcttacctaa acgggtcagg atggtcatct**

**8881 gctcctcgga ggtctcgtac agctccggaa ttaactccat catcagcttc acggagaagt**

**8941 tgtgccagcc cttgccgaag atggaggagt tggccttgcg gaactgcacc agctcgtcca**

**9001 cctgcttctg cgagaaggag ccgtcagcga actcgtgttc taaagcttct tgaatgccct**

**9061 cgcgctcggt gttcagggtc agcacgtagg ccagcttatc taaagtctcg cggtccatct**

**9121 gctcgatatc taaagtttct aaagtcttca tcttgcggta ggcctcgaag gtgtgaattt**

**9181 cggccttgcc ggacttatca atgcggtagc ccttgatgtc ggccacgtcg cacgacagca**

**9241 gcttggcgat gtacttgaac agcttggccg ggcccatcgc cttctcgttc ttcacgtagt**

**9301 tgataatttg gttcttctgc tccttcgaca gcttcttggt ctcggtcggg acggtcaggt**

**9361 tattcaggtc gttcagcagg ttgaactctt gagcggtgta ggaggccttg gcggcgcgga**

**9421 actcgtccgg gtagaaggtg cacttgccaa ttaagatgcc gaagatgtta tctaaagttt**

**9481 cgccggaggt gcggtagcgg ccgtagtcgg tgcgggactt ctcgttgccc gggccgtggt**

**9541 agtacttgcg cttgccggtc agaatctcta aatagcggtt gatgaactcg tcggtaattt**

**9601 gcgggttgaa ctcctgctgg gtttgtaaga tgcgcagggc ctcggagcga taggcggagg**

**9661 tcgggaacac gttaattaag cggtgcttct tgccgtcctt ctccacggtg aagtcgccgc**

**9721 gcagctggcc gtaggtctgg tagcgttcga gctgaatttg gcccggggtc ttggtctcca**

**9781 gctgcttgga gttctccttc acaatttggg cgtagtcgcc cacggaggag ttgccgtcgt**

**9841 cggaggcgtc atctaaatag gagatgccgc ggtgcttcac catgttcttc agggcgatga**

**9901 acagttcttc gttcgacagc tcgtcggtca ggcccttcac gcgcagctgg tacgggttca**

**9961 ggttgatgga aattttggtg aagtcggtaa ttaagccgga ctcctcgaac aggcggttca**

**10021 ggcgcacgcg gcggtgcttc ttgcgacgag ctaaacggcg accttgacgg ttggtgcggc**

**10081 gaactaaatt gttctcagct tgagcggccg ggaagatgcg cgagttcttg tggataattt**

**10141 cgccggtcac cttgttcagg atgcccacgc ccacggagcc gatgccgata gctaaaccca**

**10201 gaactaaatc ggacatctag tatttcccct ctttctctag tctcgaggct agcattatac**

**10261 ctaggactga gctagctgtc aatctagagc ggccgccacc gcggtggagc tccagctttt**

**10321 gttcccttta gtgagggtta attgcgcgct tggcgtaatc atggtcatag ctgtttcctg**

**10381 tgtgaaattg ttatccgctc acaattccac acaacatacg agccggaagc ataaagtgta**

**10441 aagcctgggg tgcctaatga gtgagctaac tcacattaat tgcgttgcgc tcactgcccg**

**10501 ctttccagtc gggaaacctg tcgtgccagc tgcattaatg aatcggccaa cgcgcgggga**

**10561 gaggcggttt gcgtattggg cgctcttccg cttcctcgct cactgactcg ctgcgctcgg**

**10621 tcgttcggct gcggcgagcg gtatcagctc actcaaaggc ggtaatacgg ttatccacag**

**10681 aatcagggga taacgcagga aagaacatgt gagatctcta cgggtcggat ttgaagtcgt**

**10741 cttggtagga ggcagcctga atggcgaatg ccgatgccct tgagagcctt caacccagtc**

**10801 agctccttcc ggtgggcgcg gggcatgact atcgtcgccg cacttatgac tgtcttcttt**

**10861 atcatgcaac tcgtaggaca gggtgccggc agcgctctgg gtcattttcg gcgaggaccg**

**10921 ctttcgctgg agcgcgacga tgatcggcct gtcgcttgcg gtattcggaa tcttgcacgc**

**10981 cctcgctcaa gccttcgtca ctggtcccgc caccaaacgt ttcggcgaga agcaggccat**

**11041 tatcgccggc atggcggccg acgcgctggg ctacgtcttg ctggcgttcg cgacgcgagg**

**11101 ctggatggcc ttccccatta tgattcttct cgcttccggc ggcatcggga tgcccgcgtt**

**11161 gcaggccatg ctgtccaggc aggtagatga cgaccatcag ggacagcttc aaggatcgct**

**11221 cgcggctctt accagcctaa cttcgatcat tggaccgctg atcgtcacgg cgatttatgc**

**11281 cgcctcggcg agcacatgga acgggttggc atggattgta ggcgccgccc tataccttgt**

**11341 ctgcctcccc gcgttgcgtc gcggtgcatg gagccgggcc acctcgacct gaatggaagc**

**11401 cggcggcacc tcgctaacgg attcaccact ccgcagaccc gccataaaac gccctgagaa**

**11461 gcccgtgacg ggcttttctt gtattatggg tagtttcctt gcatgaatcc ataaaaggcg**

**11521 cctgtagtgc catttacccc cattcactgc cagagccgtg agcgcagcga actgaatgtc**

**11581 acgaaaaaga cagcgactca ggtgcctgat ggtcggagac aaaaggaata ttcagcgatt**

**11641 tgcccgagct tgcgagggtg ctacttaagc ctttagggtt ttaaggtctg ttttgtagag**

**11701 gagcaaacag cgtttgcgac atccttttgt aatactgcgg aactgactaa agtagt**

**//**

**Supplementary Figure S3:** Fucose metabolism pathway in B. infantis ATCC15697.


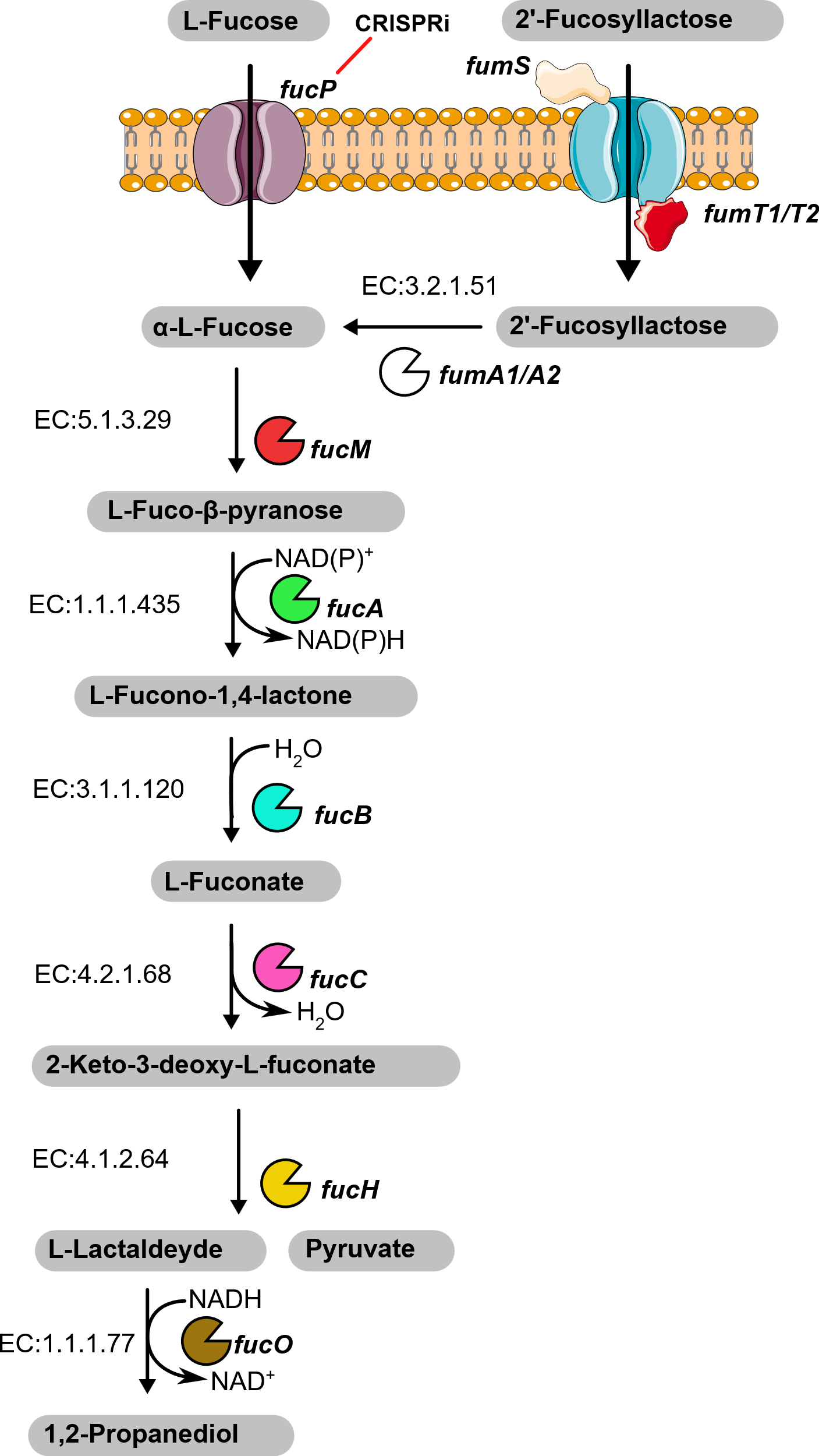


**Supplementary Table S1:** List of bacteria strains used in this study

| **Species** | | **Strain** | **Plasmid** | **Source** |
| --- | --- | --- | --- | --- |
| *Escherichia* | *coli* | EC101 |  | ^1^ |
| *Escherichia* | *coli* | XL1-Blue |  | Stratagene |
| *Escherichia* | *coli* | EC101 | pNZEM-M.blmncII | ^2^ |
| *Bifidobacterium* | *breve* | UCC2003 |  | ^3^ |
| *Bifidobacterium* | *breve* | UCC2003 | pCL007-upp3 | This study |
| *Bifidobacterium* | *breve* | UCC2003 dCas9Sth1 |  | This study |
| *Bifidobacterium* | *breve* | UCC2003 dCas9Sth1 | pNZ000_Sth1gRNA_upp1 | This study |
| *Bifidobacterium* | *breve* | UCC2003 dCas9Sth1 | pNZ000_Sth1gRNA_upp3 | This study |
| *Bifidobacterium* | *breve* | UCC2003 dCas9Sth1 | pNZ000_Sth1gRNA_upp4 | This study |
| *Bifidobacterium* | *breve* | UCC2003 dCas9Sth1 | pNZ000_Sth1gRNA_upp5 | This study |
| *Bifidobacterium* | *breve* | UCC2003 dCas9Sth1 | pNZ000_Sth1gRNA_EPS1 | This study |
| *Bifidobacterium* | *breve* | UCC2003 dCas9Sth1 | pNZ000_Sth1gRNA_EPS2 | This study |
| *Bifidobacterium* | *breve* | UCC2003 dCas9Sth1 | pNZ000_Sth1gRNA_EPS3 | This study |
| *Bifidobacterium* | *breve* | UCC2003 dCas9Sth1 | pNZ000_Sth1gRNA_rafA1 | This study |
| *Bifidobacterium* | *breve* | UCC2003 dCas9Sth1 | pNZ000_Sth1gRNA_rafA2 | This study |
| *Bifidobacterium* | *breve* | UCC2003 dCas9Sth1 | pNZ000_Sth1gRNA_rafA3 | This study |
| *Bifidobacterium* | *breve* | UCC2003 dCas9Sth1 | pNZ000_Sth1gRNA_rbsA1 | This study |
| *Bifidobacterium* | *breve* | UCC2003 dCas9Sth1nLuc |  | This study |
| *Bifidobacterium* | *breve* | UCC2003 dCas9Sth1nLuc | pNZ000_Sth1gRNA_nluc | This study |
| *Bifidobacterium* | *breve* | UCC2003 dCas9Spy |  | This study |
| *Bifidobacterium* | *breve* | UCC2003 dCas9SpynLuc |  | This study |
| *Bifidobacterium* | *breve* | UCC2003 dCas9SpynLuc | pNZ000_SpygRNA_nluc | This study |
| *Bifidobacterium* | *Animalis* subsp. *animalis* | ATCC 25527 |  | ATCC |
| *Bifidobacterium* | *animalis* subsp. *animalis* | ATCC 25527 | pCL007_upp1 | This study |
| *Bifidobacterium* | *animalis* subsp. *animalis* | ATCC 25527 | pCL007_rafA4 | This study |
| *Bifidobacterium* | *animalis* subsp. *animalis* | ATCC 25527 | pCL007_rafA5 | This study |
| *Bifidobacterium* | *longum subsp. infantis* | ATCC15697 |  | ATCC |
| *Bifidobacterium* | *longum subsp. infantis* | ATCC15697 | pCL007_upp4 | This study |
| *Bifidobacterium* | *longum subsp. infantis* | ATCC15697 | pCL007_fucP1 | This study |
| *Bifidobacterium* | *longum subsp. infantis* | ATCC15697 | pCL007_fucP2 | This study |
| *Bifidobacterium* | *longum subsp. longum* | NCIMB8809 |  | NCIMB |
| *Bifidobacterium* | *longum subsp. longum* | NCIMB8809 | pCL007_upp5 | This study |
| *Bifidobacterium* | *longum subsp. longum* | NCIMB8809 | pCL007_axuB1 | This study |
| *Bifidobacterium* | *longum subsp. longum* | NCIMB8809 | pCL007_axuB2 | This study |
| *Bifidobacterium* | *pseudocatenulatum* | DSM 20438 |  | DSM |
| *Bifidobacterium* | *pseudocatenulatum* | DSM 20438 | pCL007_upp6 | This study |

**Supplementary Table S2:** Primer sequences used in this study. Sequences in bold represent restriction sites used for cloning.

| **Primer** | **Sequence** | **Amplicon** |
| --- | --- | --- |
| CLMC_001 | GATC**GAATTC**TGGGAGACCAGAAACAAAAAAAGG | betI |
| CLMC_002 | GCACCTTGAGACACC**CTCGAG** |  |
| CLMC_003 | GATC**CTCGAG**GGTGTCTCAAGGTGC | Spy and Sth1 dCas9 |
| CLMC_004 | TTCAAGCCTACGC**GACGTC**AG |  |
| CLMC_005 | GATC**GACGTC**CCAATCAAGACCGAATACGCCAG | Non-coding region of B. breve UCC2003 CRISPR locus |
| CLMC_006 | GATC**GCGGCCGC**GCCGAGGCTGAGGCAAG |  |
| CLMC_007 | GATC**GCTCTTC**GGGGATTACAGGAGCCAGTCGTTCG | Chloramphenicol acetyltransferase |
| CLMC_008 | GATC**GCTCTTC**ACTTATGAACTTCAACAAGATTGACTTAGATAACTG |  |
| CLMC_009 | GATC**GCTCTTC**TCCCCACCAAAACCGAAATCCAC | pFREM28/29 vector backbone |
| CLMC_010 | GATC**GCTCTTC**TAAGGTGTGCTCCTTTCCCTCAC |  |
| CLMC_011 | AGATG**GCTCTTC**AGTCACTAGTAGCTTTATTGTTGTTTTTATGATTAC | ColEI origin with B. breve RM sites removed |
| CLMC_012 | AGAGT**GCTCTTC**ATAGCAATTGACGGTTATCCACAGAATCAG |  |
| CLMC_013 | AGATG**GCTCTTC**ACTACGCTCTATCCCAACTGGCTCAAG | pFREM28/29 vector backbone |
| CLMC_014 | AGAGT**GCTCTTC**AGACACGAAAAACAAGTTAAGGGATGCAG |  |
| CLMC_015 | TATAG**GCGGCCGC**ATGAAAACAACCCTGCGCGTC | 500bp internal fragment of erythromycin resistance gene |
| CLMC_016 | TATAG**TCTAGA**GAGGGCGATAACGGCAGTTG |  |
| CLMC_017 | TATAG**GAATTC**TTTACACCAACTCCTAGTAGG | Nanoluciferase expression cassette |
| CLMC_018 | TATAG**GCGGCCGC**TCAAAATGAAAAACC |  |
| CLMC_175 | GATCGAT**GAATTC**TCTGACCAGGGAAAATAGCCCTCTG | Spy and Sth1 gRNA expression cassettes |
| CLMC_176 | GATCGAT**GACGTC**GGGAGACCAGAAACAAAAAAAGGCC |  |

**Supplementary Table S3:** List of plasmids used in this study

| **Plasmids** | **Purpose** | **Source** |
| --- | --- | --- |
| pNZ44 | Gene expression in Bifidobacteria | ^4^ |
| pNZ000 | Gene expression in Bifidobacteria. *B. breve* UCC2003 RM sites removed | this study |
| pNZ000-Spy-gRNA | Cloning and expression of Spy gRNAs in Bifidobacteria | this study |
| pNZ000-Spy-gRNA-nluc | Expression of Spy gRNA targeting nanoluciferase | this study |
| pNZ000-Sth1-gRNA | Cloning and expression of Sth1 gRNAs in Bifidobacteria | this study |
| pNZ000-Sth1-gRNA-nluc | Expression of Sth1 gRNA targeting nanoluciferase | this study |
| pNZ000-Sth1-gRNA-upp1 | Expression of Sth1 gRNA targeting upp in *B. animalis* ATCC 25527 | this study |
| pNZ000-Sth1-gRNA-upp3 | Expression of Sth1 gRNA targeting upp in *B. breve* UCC2003 | this study |
| pNZ000-Sth1-gRNA-upp4 | Expression of Sth1 gRNA targeting upp in *B. infantis* ATCC15697 | this study |
| pNZ000-Sth1-gRNA-upp5 | Expression of Sth1 gRNA targeting upp in *B. longum* NCIMB | this study |
| pNZ000-Sth1-gRNA-EPS1 | Expression of Sth1 gRNA targeting Bbr_0430 in *B. breve* UCC2003 | this study |
| pNZ000-Sth1-gRNA-EPS2 | Expression of Sth1 gRNA targeting Bbr_0430 in *B. breve* UCC2003 | this study |
| pNZ000-Sth1-gRNA-EPS3 | Expression of Sth1 gRNA targeting Bbr_0430 in *B. breve* UCC2003 | this study |
| pNZ000-Sth1-gRNA-rafA1 | Expression of Sth1 gRNA targeting rafA in *B. breve* UCC2003 | this study |
| pNZ000-Sth1-gRNA-rafA2 | Expression of Sth1 gRNA targeting rafA in *B. breve* UCC2003 | this study |
| pNZ000-Sth1-gRNA-rafA3 | Expression of Sth1 gRNA targeting rafA in *B. breve* UCC2003 | this study |
| pNZ000-Sth1-gRNA-rbsA1 | Expression of Sth1 gRNA targeting rbsA in *B. breve* UCC2003 | this study |
| pFREM28 | Nonreplicating vector for insertional mutagenesis in *B. breve* | ^5^ |
| pFREM28-betI-dCas9Spy | Integration of Spy dCas9 in *B. breve* UCC2003 | this study |
| pFREM28-betI-dCas9Sth1 | Integration of Sth1 dCas9 in *B. breve* UCC2003 | this study |
| pFREM28-CAT | Nonreplicating vector for insertional mutagenesis in *B. breve* | this study |
| pFREM29 | pFREM28 with modified ColEI origin of replication | this study |
| pFREM29-CAT | pFREM29 with chloramphenicol resistance | this study |
| pFREM29-CAT-Nanoluciferase | Integration of nanoluciferase in *B. breve* UCC2003 dCas9 | this study |
| pBC1.2 | E.coli – Bifidobacteria shuttle vector | ^6^ |
| pCL001 | E.coli – Bifidobacteria shuttle vector | this study |
| pCL002 | Cloning and expression of Sth1 gRNAs in Bifidobacteria | this study |
| pCL007 | Expression of Sth1 dCas9 and gRNA in Bifidobacteria | this study |

**Supplementary Table S4:** gRNA target sequences used in this study

| gRNA | Sequence | PAM | Target Gene | Species | Strain | Figure |
| --- | --- | --- | --- | --- | --- | --- |
| nluc | ACCATGGAGATACCTCCTCG | NGG | *Nanoluciferase* | - | - | 1E |
| nluc | CACCATGGAGATACCTCCTCGG | NNAGAAG | *Nanoluciferase* | - | - | 1E |
| upp1 | AGACGAGCTCGGAGACGAGTTC | NNGGAAG | *upp* | *B. animalis* | ATCC 25527 | 2C, 2D, 5D |
| upp3 | TGACCAGTTCGGAGACGAGCTC | NNGGAAG | *upp* | *B. breve* | UCC2003 | 2C, 2D, 5D |
| upp4 | TCACAAGCTCGGAGACGAGCTC | NNGGAAG | *upp* | *B. infantis* | ATCC15697 | 2C, 2D, 5D |
| upp5 | TCACGAGTTCGGAGACGAGCTC | NNGGAAG | *upp* | *B. longum* | NCIMB 8809 | 2C, 2D, 5D |
| EPS1 | ATCAGGAGTTGCGTCCGCAATG | NNAGAAT | *Bbr0430* | *B. breve* | UCC2003 | 3D |
| EPS2 | TAACGGTGCCATCCGGAGAAGA | NNAGAAG | *Bbr0430* | *B. breve* | UCC2003 | 3D |
| EPS3 | GTTATGAGTAACGGTGCCATCC | NNAGAAG | *Bbr0430* | *B. breve* | UCC2003 | 3D |
| RafA1 | TTCTGACGGGCCAAACCACCGG | NNAGAAG | *rafA* | *B. breve* | UCC2003 | 4B, 4C |
| RafA2 | TTCTGCCCGGTGGTTTGGCCCG | NNAGAAG | *rafA* | *B. breve* | UCC2003 | 4B, 4C |
| RafA3 | CATCGTGACCAACCTGTTCGGA | NNAGAAG | *rafA* | *B. breve* | UCC2003 | 4B, 4C |
| rbsA | GCCGCCGTAGGCTCATCCAGAA | NNAGAAT | *rbsA* | *B. breve* | UCC2003 | 4E, 4F |
| fucP1 | TGGTCAGGAACGAGCCGATCAC | NNAGAAG | *fucP* | *B. infantis* | ATCC15697 | 6C, 6D |
| fucP2 | GGTTCATCGTCAGGCCCCAGGT | NNAGAAC | *fucP* | *B. infantis* | ATCC15697 | 6C, 6D |
| rafA4 | TGACGGCAACCCCGGCGTCACC | NNCCGCG | *rafA* | *B. animalis* | ATCC 25527 | 7B, 7C |
| rafA5 | GGCGTGCGTGGTCGCGTTGAAT | NNGGAAA | *rafA* | *B. animalis* | ATCC 25527 | 7B, 7C |
| axuA1 | AGTCGGCGGCACGAATGAGGAA | NNAGAAT | *axuA* | *B. longum* | NCIMB 8809 | 7E, 7F |
| axuA2 | ATTCAACGAGCCATCAGCATTC | NNAGAAG | *axuA* | *B. longum* | NCIMB 8809 | 7E, 7F |
| NT1 | AGTCTTCCGATACACAGTCGTGGAAGACCT | - | - | - | - |  |

**Supplementary Table S6:** Restriction-Modification systems in target Bifidobacteria strains

| Species | Strain | Gene | Target Sequence | Count in pCL007 |
| --- | --- | --- | --- | --- |
| *B. animalis* | ATCC 25527 | Ban25527ORF4345P | GACGAG | 15 |
| *B. breve* | UCC2003 | Bbr_1118 | CTGCAG | 1 |
|  |  | Bbr_1120 | RTCGAY | 2 |
|  |  | Bbr_215 | GGCGCC | 2 |
| *B. infantis* | ATCC15697 | Blon_91 | GGCGCC | 2 |
|  |  | Blon_289 | CTGCAG | 1 |
|  |  | Blon_1146 | GTCGAC | 0 |
|  |  | Blon_1324 | GAGGAC | 5 |
| *B. longum* | NCIMB8809 | B8809_1355 | GATNNNNNTGCC | 2 |
|  |  | B8809_606 | CCWGG | 6 |
| *B. pseudocatenulatum* | DSM20438 | Bps20438_772 | GACNNNGTC | 1 |

**References**

1. Law, J. *et al.* A system to generate chromosomal mutations in Lactococcus lactis which allows fast analysis of targeted genes. *Journal of Bacteriology* **177**, 7011–7018 (1995).

2. O’Callaghan, A., Bottacini, F., O’Connell Motherway, M. & van Sinderen, D. Pangenome analysis of Bifidobacterium longum and site-directed mutagenesis through by-pass of restriction-modification systems. *BMC Genomics* **16**, 832 (2015).

3. Mazé, A., O’Connell-Motherway, M., Fitzgerald, G. F., Deutscher, J. & van Sinderen, D. Identification and Characterization of a Fructose Phosphotransferase System in Bifidobacterium breve UCC2003. *Applied and Environmental Microbiology* **73**, 545–553 (2007).

4. McGrath, S., Fitzgerald, G. F. & van Sinderen, D. Improvement and optimization of two engineered phage resistance mechanisms in Lactococcus lactis. *Appl Environ Microbiol* **67**, 608–616 (2001).

5. Hoedt, E. C. *et al.* Broad Purpose Vector for Site-Directed Insertional Mutagenesis in Bifidobacterium breve. *Front. Microbiol.* **12**, (2021).

6. Álvarez-Martín, P., Belén Flórez, A., Margolles, A., del Solar, G. & Mayo, B. Improved Cloning Vectors for Bifidobacteria, Based on the Bifidobacterium catenulatum pBC1 Replicon. *Applied and Environmental Microbiology* **74**, 4656–4665 (2008).
